# The Stress-Induced Transcription Factor NR4A1 Adjusts Mitochondrial Function and Synapse Number in Prefrontal Cortex

**DOI:** 10.1523/JNEUROSCI.2793-17.2017

**Published:** 2018-02-07

**Authors:** Freddy Jeanneteau, Christian Barrère, Mariska Vos, Carlie J.M. De Vries, Claude Rouillard, Daniel Levesque, Yann Dromard, Marie-Pierre Moisan, Vanja Duric, Tina C. Franklin, Ronald S. Duman, David A. Lewis, Stephen D. Ginsberg, Margarita Arango-Lievano

**Affiliations:** ^1^Département de Neuroscience et Physiologie, Institut de Génomique Fonctionnelle, Institut National de la Santé et de la Recherche Médicale, Centre National de Recherche Scientifique, Université de Montpellier, Montpellier, 34090 France,; ^2^Department of Medical Biochemistry, Academic Medical Center, University of Amsterdam, 1012 WX Amsterdam, The Netherlands,; ^3^Département de Psychiatrie et Neuroscience, Université Laval, Québec City, Québec G1V 0A6, Canada,; ^4^Faculté de Pharmacie, Université de Montréal, Montréal, Québec H3T 1J4, Canada,; ^5^Nutrition and Integrative Neurobiology, Institut National de la Recherche Agronomique, Université de Bordeaux, 33076 Bordeaux, France,; ^6^Department of Physiology and Pharmacology, Des Moines University, Des Moines, Iowa 50312,; ^7^Department of Psychiatry and Neurobiology, Yale University, New Haven, Connecticut 06520,; ^8^Department of Psychiatry, University of Pittsburgh, Pittsburgh, Pennsylvania 15260,; ^9^The Nathan S. Kline Institute for Pyschiatric Research, Orangeburg, New York 10962, and; ^10^Department of Psychiatry, Neuroscience & Physiology, NYU Langone Medical Center, New York, New York 10016

**Keywords:** dendritic spines, mitochondria, prefrontal cortex, pyramidal neurons, stress

## Abstract

The energetic costs of behavioral chronic stress are unlikely to be sustainable without neuronal plasticity. Mitochondria have the capacity to handle synaptic activity up to a limit before energetic depletion occurs. Protective mechanisms driven by the induction of neuronal genes likely evolved to buffer the consequences of chronic stress on excitatory neurons in prefrontal cortex (PFC), as this circuitry is vulnerable to excitotoxic insults. Little is known about the genes involved in mitochondrial adaptation to the buildup of chronic stress. Using combinations of genetic manipulations and stress for analyzing structural, transcriptional, mitochondrial, and behavioral outcomes, we characterized NR4A1 as a stress-inducible modifier of mitochondrial energetic competence and dendritic spine number in PFC. NR4A1 acted as a transcription factor for changing the expression of target genes previously involved in mitochondrial uncoupling, AMP-activated protein kinase activation, and synaptic growth. Maintenance of NR4A1 activity by chronic stress played a critical role in the regressive synaptic organization in PFC of mouse models of stress (male only). Knockdown, dominant-negative approach, and knockout of *Nr4a1* in mice and rats (male only) protected pyramidal neurons against the adverse effects of chronic stress. In human PFC tissues of men and women, high levels of the transcriptionally active NR4A1 correlated with measures of synaptic loss and cognitive impairment. In the context of chronic stress, prolonged expression and activity of NR4A1 may lead to responses of mitochondria and synaptic connectivity that do not match environmental demand, resulting in circuit malfunction between PFC and other brain regions, constituting a pathological feature across disorders.

**SIGNIFICANCE STATEMENT** The bioenergetic cost of chronic stress is too high to be sustainable by pyramidal prefrontal neurons. Cellular checkpoints have evolved to adjust the responses of mitochondria and synapses to the buildup of chronic stress. NR4A1 plays such a role by controlling the energetic competence of mitochondria with respect to synapse number. As an immediate-early gene, *Nr4a1* promotes neuronal plasticity, but sustained expression or activity can be detrimental. NR4A1 expression and activity is sustained by chronic stress in animal models and in human studies of neuropathologies sensitive to the buildup of chronic stress. Therefore, antagonism of NR4A1 is a promising avenue for preventing the regressive synaptic reorganization in cortical systems in the context of chronic stress.

## Introduction

Stress, especially when it is chronic and uncontrollable, produces depressive-like phenotypes and cognitive impairment in animal models (Krishnan and Nestler, 2011; McEwen and Morrison, 2013; McKlveen et al., 2013). Chronic stress impacts neuronal plasticity throughout the brain in part through excessive levels of glutamate and corticosterone (CORT) secretion that remodel dendritic territories in a manner that alters their functional properties (Pittenger and Duman, 2008; Popoli et al., 2011; Myers et al., 2014). Excessive levels of CORT impair neuronal sensitivity to serotonin and neurotrophins, resulting in reduced synapse number and neurotransmission (van Riel et al., 2003; Arango-Lievano et al., 2015). In hippocampus and cortex, chronic stress and CORT inhibit long-term potentiation (Kim and Diamond, 2002; Goldwater et al., 2009), enhance long-term depression (Xu et al., 1997), and produce dendritic atrophy, spine loss, and eventually cell death (Sapolsky et al., 1985; Radley et al., 2006; Liston et al., 2013; McEwen and Morrison, 2013).

Responses to acute and chronic stress are both considered adaptive to prepare for current and future demands, as they are paramount for survival (Karatsoreos and McEwen, 2011; Myers et al., 2014). The typical inverted U-shaped relationship between stress/CORT inputs and functional outputs (mitochondrial/synaptic/behavioral) indicates that neuronal perceptions and responses to external signals are flexible to fit with network demands (Picard and McEwen, 2014; Sapolsky, 2015; Jeanneteau and Arango-Lievano, 2016). Neurons use their genomic and epigenomic arsenal to adapt their metabolism in accordance with their connectivity and network activity (Attwell and Laughlin, 2001; Gray et al., 2017). For instance, repetitive trains of excitation during seizures cause mitochondrial dysfunction leading, eventually, to neuronal death (Tang and Zucker, 1997). Modeling studies indicate that synaptic depotentiation is desirable to support neuronal survival when energetic stores are limited. Synaptic scaling changes mitochondrial functions in response to excessive/depressed glutamatergic excitation (Bindokas et al., 1998). Negative feedback mechanisms may have evolved to suppress synapse potentiation that would drain energetic stores upon glutamatergic overexcitation.

Checkpoints could lie in the genes that control signaling loops between mitochondria and synapses (Li et al., 2004; Jeanneteau and Arango-Lievano, 2016). Hundreds of genes are induced/repressed in response to neuronal activity and stress, and, remarkably, each gene has a fine homeostatic pattern of expression (Valles et al., 2011; Datson et al., 2012; McEwen et al., 2015). These newly transcribed genes play important roles in many aspects of the adaptive abilities of the brain through the regulation of neuronal metabolism, dendritic growth, and synapse remodeling. We focused on *Nr4a1* (*Nur77/NgfI-B*) because it shuffles among mitochondria, cytosol, and nucleus to modify metabolism (Zhao and Bruemmer, 2010; Pawlak et al., 2015), synaptic plasticity (Bridi and Abel, 2013; Chen et al., 2014), and cognition (Hawk and Abel, 2011; McNulty et al., 2012). NR4A1 has the necessary attributes to adapt mitochondrial functions and synaptic activity in the context of stress, as it is an activity-dependent immediate early gene responding to a variety of stressors and sensory stimuli (Chen et al., 2014; Helbling et al., 2014). Using complementary genetic approaches, we provide evidence for a causal role of NR4A1 in mediating stress/CORT-elicited dendritic spine loss in prefrontal cortex (PFC). NR4A1 acted as a transcription factor to change the expression of target genes previously involved in wasting the mitochondrial energetic budget and activating the AMP-activated protein kinase (AMPK) catabolic pathway (Steinberg and Kemp, 2009; Weisová et al., 2012). Inappropriate processing of this pathway during chronic stress, as opposed to acute stress, may lead to responses of mitochondrial function and synaptic connectivity that do not match environmental demand. As a result, stress-induced circuit malfunction between PFC and other brain regions may be a pathological feature across disorders (Sampath et al., 2017). This prompted us to validate results from animal studies to human studies of major depressive disorders (MDDs) and Alzheimer's disease (AD), as neuropathology in both diseases is aggravated by stress involving synaptic loss in PFC, disrupted CORT levels, and cognitive impairment (Lupien et al., 2009; Sotiropoulos et al., 2011; Heim and Binder, 2012).

## Materials and Methods

All experiments were performed in accordance with the Directive by the Council of the European Communities (86/609/EEC) and approved protocols (00651.01) following institutional guidelines for the care and use of laboratory animals.

### 

#### 

##### Reagents.

Corticosterone, STO609 (CAMKK inhibitor), A769662 (AMPK activator), glutamate, oligomycin, and carbonyl cyanide-4-(trifluoromethoxy)phenylhydrazone (FCCP) are from Sigma-Aldrich S.A.R.L. Doxycycline is from Clontech. Brain-derived neurotrophic factor (BDNF) is from Abnova. Tetramethylrhodamine methyl ester (TMRM) is from Santa Cruz Biotechnology. The antibodies used were as follows: cyto-NR4A1 (ABIN460855, antibodies-online.com); pan-NR4A1 (E6, Santa Cruz Biotechnology); and anti-human NR4A1 (D63C5), phospho (p)-NR4A1 (S350-P), ubiquitin (P4D1), P190A, phospho-acetyl-CoA carboxylase (ACC; S79-P), AMPK and phospho-AMPK (T172-P), HDAC2, and S6 (Cell Signaling Technology). Actin is from Sigma-Aldrich S.A.R.L.; FKBP51 and HSP90 are from BD Biosciences-Europe; GAPDH is from Thermo Fisher Scientific; and GFP and Drebrin (M2F6) are from Abcam. RFP is from Rockland Immunochemicals; PSD-95 is from NeuroMab; synaptophysin is from Thermo Fisher Scientific; and NR1 (ab1516) and NR2 (ab1548) are from Merck Millipore.

##### Rodent models.

Thy1-YFP transgenic mice [B6.Cg-Tg(Thy1-YFP)HJrs/J; Feng et al., 2000] and NR4A1 knock-out (KO) mice (B6;129S2-*Nr4a1^tm1Jmi^*/J; Lee et al., 1995) were grown on a pure C57BL/6 background. Time-pregnant CD1 mice (Janvier Labs) were used for *in utero* electroporation experiments. Frozen brain tissues from Nr4a1-deficient rats (Nr4a1m1Mcwi) grown on a Fawn-Hooded Hypertensive background [Transposagen Biopharmaceuticals and the National Institutes of Health (NIH) Rat Knockout Consortium Program (http://www.transposagenbio.com/knock-out-rat-consortium)]. All animals were allowed *ad libitum* access to food and water and were maintained on a 12 h light/dark cycle. Males were used in all protocols. Chronic unpredictable stress includes one of the following daily random stressors for 10 consecutive days from postnatal day 21 (P21): wet bedding, no bedding, food deprivation, crowded cage, 2 or 6 h restrain, forced swim, shaking, 24 h light cycle, and tail suspension. Doxycycline (2 mg/ml) was administered via the drinking water and refreshed every 3 d.

##### Tail suspension test.

One-month-old mice were subjected to a single tail suspension test on the day before being killed. For habituation, the mouse tail was taped 5 min before suspension to a hook located in a 56 × 40 × 33 cm dark box. Frontal visual inspection of immobility postures was measured using a timer. Slight movements of hindlimbs only were also considered as immobility, as described previously (Can et al., 2012). Immobility in the tail suspension test (TST) is a typical measure of behavioral despair modified by long-term administration of CORT and chronic stress (Krishnan and Nestler, 2011).

##### *In utero* electroporation.

One microgram of DNA was injected into the ventricle at embryonic day 15 (E15) on CD1 mouse embryos and electroporated (NEPA21 Super Electroporator, NEPAGENE; 30 V; pulse ON, 50 ms; pulse OFF, 950 ms; 5 pulses) as described previously (Arango-Lievano et al., 2015). Mice were anesthetized with 4% isoflurane/oxygen and maintained at 1.5–2% isoflurane (Abbott Laboratories) throughout surgery using TEC3N (Anesteo). Mice received preemptive analgesia with lidocaine (Xylovet; 3.5 mg/kg at incision site). A subcutaneous injection of the analgesic buprenorphine (Buprecare; 0.05 mg/kg) was administered postsurgery and the next day. The expression of transgenes in experimental animals generated by *in utero* electroporation was stable in adolescents but faded before reaching adulthood (Arango-Lievano et al., 2016). Due to this technical reason, adolescent mice were used throughout the study.

##### Microdissection and lysate preparation.

Animals were anesthetized with pentobarbital (for mice, 50 mg/kg; for rats, 100 mg/kg, i.p.; Ceva Santé Animale) before decapitation. Cortical biopsy samples were punched out of fresh mouse brain sections (200 μm) on ice using a punch set and snap frozen in liquid nitrogen. Tissue homogenates were lysed in 10 mm Tris-HCl, pH 8.0, 150 mm NaCl, 1 mm EDTA, 10% glycerol, 1% NP-40, 0.1% SDS, 0.1% Triton X-100 complemented with protease inhibitors, 1 mm Na_3_VO_4_, 10 mm NaF, and 10 nm calyculin A. Protein samples for Western blot analysis were cleared from debris by centrifugation (14,000 rpm for 10 min).

##### Cell culture and lysis.

Time-pregnant Sprague Dawley rats (Janvier Labs) were killed by narcosis to prepare primary E18 cortical neurons cultured on glass coverslips coated with poly-d-lysine, and maintained in Neurobasal medium containing B27 supplement, 0.5 mm l-glutamine, 5-fluorouridine, and uridine (10 μm each; Thermo Fisher Scientific) as previously described (Lambert et al., 2013). HEK293 cells were grown in DMEM containing 10% FBS. Cell lysates were prepared in 10 mm Tris-HCl, pH 8.0, 150 mm NaCl, 1 mm EDTA, 10% glycerol, 1% NP-40, 0.1% SDS plus protease inhibitors, 1 mm Na_3_VO_4_, 10 nm calyculin A, and 10 mm NaF and cleared (14,000 rpm for 10 min). Primary cortical cultures were grown for 3 weeks (DIV21) before analysis of spine morphology and biochemistry.

##### DNA and transfections.

Luciferase (Luc)–reporter constructs consist of a minimal POMC inactive promoter used as inactive control and 3× tandem NurRE-POMC fusion (a gift from J. Drouin, Montreal Clinical Research Institute, Montreal, QC, Canada). Short hairpin RNA (shRNA) plasmids against murin *nr4a1* and scramble (sc) sequences are from GeneCopoeia (Tebu-Bio). NR4A1 cDNA (a gift from P. Tontonoz, UCLA, Los Angeles, CA) was subcloned in pSLIK-venus under the control of a doxycycline-inducible promoter (ATCC). *In vitro* electroporation of plasmid constructs of primary cortical neurons were performed with the AMAXA System according to manufacturer instructions (Lonza). HEK293 cells were transfected with Lipofectamine 2000 (Thermo Fisher Scientific).

##### Mutagenesis.

NR4A1 mutants [R337A, ΔNLS (nuclear localization sequence), S340A, and S350A] were made by site-directed mutagenesis using QuikChange (Agilent Technologies). Deletion mutant ΔAF1 was generated by PCR and subcloned into pSLIK-venus. All constructs were verified by sequencing.

##### NR4A1 transcriptional activity.

NurRE-Luc together with renilla plasmids (10:1 ratio) were electroporated in primary neurons or in layer II/III cortical neurons *in vivo* together with GFP (10:1:2 ratio). Luciferase/renilla activities were assessed with dual Luciferase reporter assay according to manufacturer instructions (Promega) from extracts of primary neurons or GFP-positive biopsies of cortex microdissected under a fluorescence microscope (MZ16F Fluorescence Microscope, Leica Microsystems) from P31 mice anesthetized with pentobarbital (50 mg/kg, i.p.) and perfused at a rate of 3 ml/min through the ascending aorta with 30 ml of ice-cold 0.9% NaCl before decapitation.

##### Nuclear fractionation.

To purify nuclear extracts, neurons were rinsed in ice-cold PBS and incubated for 15 min on ice with buffer A (10 mm HEPES-KOH, pH 7.9, 10 mm KCl, 0.1 mm EDTA, 0.1 mm EGTA, and 1 mm DTT with protease and phosphatase inhibitors) before harvesting, 0.5% NP-40 was added to the cells for 3 min on ice, and lysates were centrifuged for 1 min at 13,000 rpm. The resulting supernatant was stored as the cytoplasmic fraction, and the pellet was further rinsed in 1 ml of buffer A. The pellet was vortexed for 20 min in 50 ml of buffer B (20 mm HEPES-NaOH, pH 7.9, 0.4 mm NaCl, 1 mm EDTA, and 1 mm EGTA with protease and phosphatase inhibitors) and cleared by centrifugation for 10 min at 13,000 rpm. The resulting supernatant was collected as the nuclear fraction, and purity was assessed using markers HSP90 and HDAC2.

##### Immunoprecipitation.

Protein concentrations were measured with the Bradford assay against BSA standards (Thermo Fisher Scientific). Polyclonal antibodies against pan-NR4A1 (E6, Santa Cruz Biotechnology) were used for immunoprecipitation with protein A-conjugated magnetic beads (Thermo Fisher Scientific) and Western blot for detecting Ubiquitin (P4D1, Cell Signaling Technology), cyto-NR4A1 (ABIN460855, antibodies-online.com) and pan-NR4A1 by chemiluminescence (ECL, GE Healthcare Life Sciences). Densitometric analysis of grayscale images was performed with ImageJ (NIH).

##### TMRM fluorescence live imaging.

Imaging of TMRM (50 μm), quenching mode (in 120 mm NaCl, 3.5 mm KCl, 0.4 mm KH_2_PO_4_, 15 mm glucose, 1.2 mm CaCl_2_, 5 mm NaHCO_3_, 1.2 mm Na_2_SO_4_, and 20 mm HEPES, pH 7.4) with an inverted fluorescence microscope (model IX70, Olympus) coupled with a Coolsnap HQ Camera (Roper Scientific SARL) from primary cortical neurons electroporated with the indicated construct and seeded on poly-d-lysine-coated Ibidi glass bottom dishes (BioValley). GFP-positive cells were identified prior to recording changes of TMRM fluorescence over time (Δ*F* = *F* − *F*_0_/*F*_0_ × 100, where *F* is fluorescence intensity at any time point and *F*_0_ is baseline fluorescence) by illumination at 555 nm and detection at 570 nm (Metafluor software, Molecular Devices). Background intensity was subtracted from whole-cell intensity, and *F*_0_ was measured as the average normalized fluorescence emitted during the 120 s before glutamate treatment. Images were captured every 5 s. For quantification, we extracted the average of the last 3 min of each epoch (100 μm glutamate, washout, 1 μm oligomycin, and 10 μm FCCP) due to the lag of time during perfusion of drugs or a rinse with culture medium. Statistical comparisons between groups reported differences during epochs (glutamate: *N* = 16 GFP; *N* = 27 NR4A1; *N* = 7 ΔAF1; washout: *N* = 16 GFP; *N* = 27 NR4A1; *N* = 7 ΔAF1; oligomycin: *N* = 10 GFP; *N* = 10 NR4A1; *N* = 7 ΔAF1; FCCP: *N* = 10 GFP; *N* = 10 NR4A1; *N* = 7 ΔAF1).

##### ATP concentrations.

Whole-cell ATP levels were monitored using the ATP Bioluminescence Assay Kit HSII according to manufacturer instructions (Sigma-Aldrich S.A.R.L.). ATP levels were normalized to the total amount of proteins dosed with Bradford assay against BSA standards (Thermo Fisher Scientific).

##### Golgi staining and dendritic spine studies.

**Figure 1.**
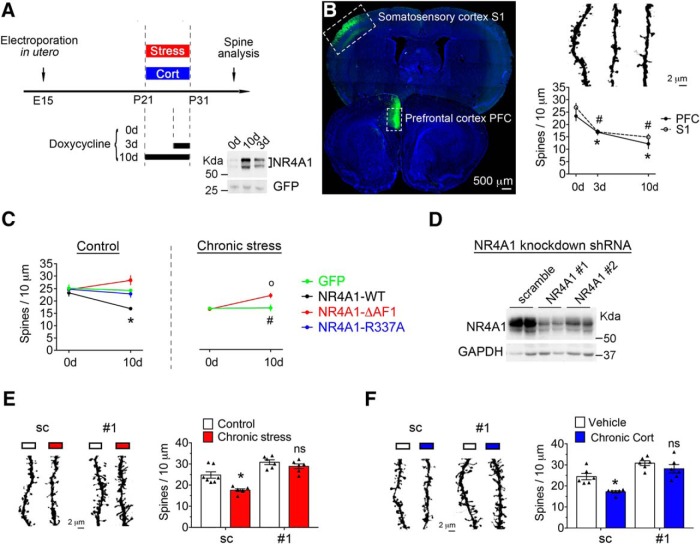
Chronic activation of the glucocorticoid stress pathway required NR4A1 for dendritic spine attrition in pyramidal neurons of mouse cortex. ***A***, Experimental timeline: doxycycline-inducible NR4A1 construct (with GFP reporter) was electroporated *in utero*, and doxycycline was administered in drinking water for the indicated days between P21 and P31. During this period, mice were exposed either to daily unpredictable stress or CORT injections (15 mg/kg). See extended data Figure 1-1. ***B***, Spine density at apical tuft dendrites of cortical pyramidal L2/3 neurons of S1 and PFC electroporated with NR4A1 (mean ± SEM of *N* = 5 mice/group, ANOVA *post hoc* Bonferroni's test comparing 0 vs 3 d: #*p* = 0.0168; *p < 0.0001; 0 vs 10 d: *,*#p* < 0.0001). See extended data Figure 1-2. ***C***, Spine density at apical tuft dendrites of cortical pyramidal L2/3 neurons electroporated with NR4A1 constructs in S1. Mice were either reared in standard homecage conditions or exposed to chronic unpredictable stress. Mean ± SEM of *N* = 5–7 mice/group; ANOVA *post hoc* Bonferroni's test: NR4A1 vs GFP, *p = 0.0031; stress vs controls; #*p* = 0.0015; interaction of stress and DNA construct expression *post hoc* Bonferroni's test ΔAF1, o*p* = 0.0018. ***D***, Knockdown of transfected NR4A1 in HEK cells with shRNA#1 and #2 compared with sc sequence. ***E***, Effect of NR4A1 knockdown on stress-induced spine loss at apical tuft dendrites of pyramidal L2/3 neurons in S1 (mean ± SEM of *N* = 6 mice/group; ANOVA *post hoc* Bonferroni's test comparing stress and controls in scramble group, *p = 0.0018). See extended data Figure 1-3. ***F***, Effect of NR4A1 knockdown on CORT-induced spine loss at apical tuft dendrites of pyramidal L2/3 neurons in S1 (mean ± SEM of *N* = 6–7 mice/group; ANOVA *post hoc* Bonferroni's test comparing stress and controls in scramble group, *p = 0.0017). See extended data Figure 1-3.

**Figure 2.**
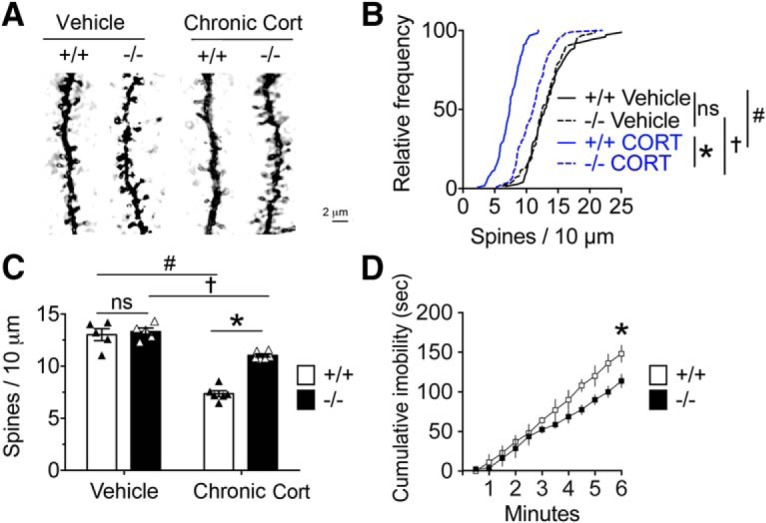
Knockout of NR4A1 protected against adverse effects of chronic CORT injections. ***A***, Apical tuft dendrites of Golgi-impregnated pyramidal L2/3 neurons in PFC from NR4A1 KO mice exposed to vehicle or CORT daily injections between P21 and P31. ***B***, Distribution of dendritic spine territories at the apical tuft of the layer 2/3 neurons in PFC (dendrites for vehicle groups: *N* = 72 +/+; *N* = 61 −/−; dendrites for CORT groups: *N* = 110 +/+; *N* = 107 −/−). Comparison with K-S test: *,#*p* < 0.0001; †*p* = 0.0016. ***C***, Group effects on spine density (mean ± SEM of *N* = 5–6 mice/group; ANOVA *post hoc* Bonferroni's test comparing the effect of genotype and CORT treatment: *,#*p* < 0.0001; †*p* = 0.0066). ***D***, Immobility time in the TST of CORT-injected NR4A1 KO mice compared with wild-type littermates (mean ± SEM of *N* = 5–6 mice/group; ANOVA *post hoc* Sidak's *t* test comparing total immobility time between genotypes, *p < 0.05).

Animals were anesthetized with pentobarbital (50 mg/kg, i.p.) and perfused at a rate of 3 ml/min through the ascending aorta with 30 ml of 0.9% NaCl prior decapitation. The FD-Rapid GolgiStain Kit was used according to manufacturer instructions (FD Neurotechnologies), and labeled brain slices were imaged with transmitted light on an AxioImager Z1 (Carl Zeiss) equipped with a 100× oil-immersion objective. Fluorescence images from blinded groups were taken on an LSM510 laser-scanning confocal microscope (pinhole set to 1 airy unit; Carl Zeiss) equipped with 63× Plan-Neofluor NA1.3 oil-immersion objective and digital zoom 8. *Z*-stack images were processed using ImageJ (NIH). Laser excitation, fluorescence emission capture, and the pinhole were held constant throughout the study. Dendritic segments included in the analysis met the following criteria: (1) segments must be parallel or at acute angles relative to the coronal surface of sections to allow unambiguous identification of spines; (2) segments had no overlap with other branches; (3) dendritic segments from apical tuft were imaged in cortical layer 1 distant from ∼100 μm from pyramidal neuronal soma in layer 2; and (4) dendritic segments from apical tuft were imaged in cortical layer 1 at a distance of ∼200 μm from pyramidal neuronal soma in layer 3. The total length of dendrites analyzed for dendritic spine density exceeded 100 μm/mouse, which exceed 500 μm/group, depending on the number of animals. Not more than two dendritic segments per cell (for which branch order was not recorded) were scored in a total of 10–15 cells/per group. Thin, mushroom, and stubby spines were counted as one unique category, and filopodia were disregarded because they were extremely rare at P31. A total exceeding 1000 dendritic spines from at least 20–30 dendritic segments were counted per group, which depended on the number of animals per group (*N* is indicated in the figure legends). The number of dendritic spines enumerated at pyramidal cortical neurons depends on methodology, species, and age, as previously reported (Cerqueira et al., 2007; Radley et al., 2008; Bloss et al., 2011; Anderson et al., 2016). For example, the range is 15–30 spines/10 μm at apical tuft dendrites of PFC in adolescent mice monitored by fluorescence confocal microscopy in Figure 1 and as described previously (Radley et al., 2008; Swanson et al., 2013), whereas it is 6–13 spines/10 μm at apical tuft dendrites of PFC in adolescent mice monitored in Golgi-Cox-impregnated neurons imaged by transmitted light microscopy in Figure 2 and as described previously (Cerqueira et al., 2007). *In vitro* after 3 weeks in culture, the range is three to seven dendritic spines per 10 μm in primary cortical neurons in Figure 6*F* and as described previously (Arango-Lievano et al., 2015).

##### Histochemistry.

Animals were anesthetized with pentobarbital (50 mg/kg, i.p.) and perfused at a rate of 3 ml/min through the ascending aorta with 30 ml of 0.9% NaCl, followed by 30 ml of 4% ice-cold PFA. Brains were harvested and postfixed for 2 h and equilibrated in 30% sucrose (Sigma-Aldrich S.A.R.L.). Free-floating coronal sections rinsed in PBS were blocked in 5% normal goat serum, 5% normal horse serum, PBS, and 0.1% Triton X-100 for 2 h at 25°C. Primary antibodies (GFP, 1:3000; RFP, 1:2000; pan-NR4A1, 1:400) were incubated for 2 d at 4°C, and secondary antibodies (1:2000; Thermo Fisher Scientific) for 2 h at 25°C. Cultured cells were fixed in 4% PFA, 20% sucrose in PBS for 10 min at 25°C. After quenching the fixative with 50 mm NH_4_Cl in PBS, cells were permeabilized with 0.1% Triton X-100 in PBS for 3 min; blocked with 10% goat serum, 2% BSA, and 0.25% fish skin gelatin in TBS for 30 min; and then incubated with antibodies for 3 h in blocking solution at room temperature. Cells were washed in TBS, 0.25% fish skin gelatin, and mounted in mowiol (Sigma-Aldrich S.A.R.L.). Antibodies against p-NR4A1 and cyto-NR4A1 did not work for immunostaining. This is why quantitative analyses were performed by Western blot. NR4A1 antibodies recognized two major bands at 60 and 75 kDa, which disappear in KO cultures (Chen et al., 2014). Post-translational modifications like phosphorylation, ubiquitination, and sumoylation explain, in part, the shift of molecular weights (Hazel et al., 1991; Chen et al., 2014; Zhang et al., 2017).

##### RNA extraction.

Total RNA from primary neurons was extracted with TRIzol (Thermo Fisher Scientific), and cDNA was synthesized from 1 μg of RNA using the First-Strand cDNA Synthesis Kit for Real-Time PCR (USB, Thermo Fisher Scientific) and random primer mix (USB, Thermo Fisher Scientific).

##### Quantitative PCR.

The Hot-start SYBR Green PCR kit (Qiagen) was used in 10 μl reactions containing 2.5 μl of cDNA and a 100 nm primer mixture. The rodent-specific primers used are as follows: *drp1*, 5′-TGATGGGAAGGGTTATTCCA-3′ and 5′-TGGCCAGAGATGGGTACTTC-3′; *mfn1*, 5′-TCGCAGTCAGCAGTGAAAAC-3′ and 5′-TGCCACGTTTACTGAGTCCA-3′; *mfn2*, 5′-AGGAAATTGCTGCCATGAAC-3′ and 5′-TGTTGAGTTCGCTGTCCAAC-3′; *opa1*, 5′-GGACCCAAGAGCAGTGTGTT-3′ and 5′-GGTTCTTCCGGACTGTGGTA-3′; *fis1*, 5′-GCCTGGTTCGAAGCAAATAC-3′ and 5′-CACGGCCAGGTAGAAGACAT-3′; *ucp2*, 5′-ACAAGACCATTGCACGAGAG-3′ and 5′-ATGAGGTTGGCTTTCAGGAG-3′; *ucp4*, 5′-TCTCACAAAAACCCGACTCC-3′ and 5′-ACCATCCGACCTCCAGAGTA-3′; *ucp5*, 5′-GGAATGCTGGGAGACACAAT-3′ and 5′-GTCCCACTATTGCCCTCTGA-3′; *gapdh*, 5′-cctgcaccaccaactgcttag-3′ and 5′-ctgtggtcatgagcccttcc-3′; human *nr4a1.3*, 5′-tctatgtcctcgccttggtt-3′ and 5′-attatcccgtctgccttcag-3′; and human *tubb*, 5′-cagggcttccagctgacccactc-3′ and 5′-gtgagggcatgacgctgaaggtg-3′. Quantitative PCR (qPCR) was performed with an ABI 7900 Instrument (Thermo Fisher Scientific), followed by melt–curve analysis. Fold changes in gene expression were calculated using the ΔΔCt (Ct = cycle number at threshold) analytical method, which includes normalization against housekeeping gene human *TUBB* (encoding α-tubulin) or rodent *GAPDH*.

##### Human studies: AD.

Frozen tissues corresponding to Brodmann areas 9–10 (medial anterior prefrontal cortex) from 24 case patients in whom AD was diagnosed (12 with moderate AD: 7 females/5 males; 12 with severe AD: 8 females/4 males) and 17 healthy cognitively normal control subjects (6 females/11 males) were obtained from the Rush Memory and Aging Project and Religious Orders Study, the University of Pennsylvania Brain Bank (Center for Neurodegenerative Disease Research, Philadelphia, PA), the Harvard Brain Bank (Harvard Brain Tissue Resource Center, Belmont, MA), and the Emory Brain Bank (Center for Neurodegenerative Disease, Atlanta, GA) as previously described (Ginsberg et al., 2010a,b; Arango-Lievano et al., 2016) with a premortem clinical diagnosis and cognitive assessment scores collected within 1 year before death using the Mini-Mental State Examination (MMSE; Folstein et al., 1975). A board-certified neuropathologist blinded to the clinical diagnosis performed a neuropathological diagnosis based on established criteria (Braak and Braak, 1991; Hyman and Trojanowski, 1997). Exclusion criteria included argyrophilic grain disease, frontotemporal dementia, Lewy body disease, mixed dementias, Parkinson's disease, and stroke. Subject groups matched as closely as possible for age and postmortem interval (PMI; for details, see Fig. 9-1). Medications for persons during life were not accessible as per Health Insurance Portability and Accountability Act of 1996 (HIPAA) guidelines for a pathological study. Informed consent was obtained for all subjects. This study is performed under the guidelines of the Nathan S. Kline Institute and the NYU Langone Medical Center. Tissue samples were processed as described previously (Ginsberg et al., 2000; Counts et al., 2004), and Western blots repeated five times for each sample to ascertain reproducibility and minimize experimental bias. Experimentalists were blind to the groups. Digitized data were measured by optical densitometric analysis with NIH ImageJ, and background was subtracted and normalized to GAPDH levels.

##### Human studies: MDD.

Frozen tissues corresponding to Brodmann area 9 (medial prefrontal cortex) from 27 depressed subjects (14 females/13 males) and 27 psychiatrically healthy control subjects (14 females/13 males) matched for sex and as closely as possible for age and PMI were obtained from the University of Pittsburgh Brain Tissue Donation Program (Pittsburgh, PA). All tissue samples were obtained at autopsy following consent from the next of kin. All depressed subjects met the diagnostic criteria for MDD according to the *Diagnostic and Statistical Manual of Mental Disorders*, fourth edition (American Psychiatric Association, 1994). Control subjects did not meet the criteria for an axis I disorder at any time in their lives. Subject groups did not differ in mean age, PMI, brain pH, RNA integrity number, or tissue storage time (for details, see Fig. 8-1). Informed consent was obtained for all subjects. Details of medications of persons during life were not accessible as per HIPAA guidelines for a pathological study. This study is performed under the guidelines of the Yale University School of Medicine. Total RNA (500 ng) extracted from human PFC was reverse transcribed into cDNA using random hexamer primer mix and SuperScriptIII qRT-PCR Kit (Thermo Fisher Scientific; Duric et al., 2010). Experimentalists were blinded to the subject groups. A combination of factors accounted for the reduced number of samples (*N* = 25 control subjects and *N* = 24 MDDs) in the qPCR analysis (e.g., low signal strength or statistical outliers based on SD).

##### Statistics.

Data were compared using a two-tailed Student's *t* test, correlated with Pearson *r* for comparing two sets of variables. Cumulative distribution comparing two variables was performed with Kolomogorov–Smirnov (K-S) test. We used a factorial ANOVA to compare multiple groups, using stress status, CORT treatment, genotypes, NR4A1 constructs, doxycycline treatment serving as independent factors, followed by *post hoc* pairwise comparison with appropriate tests (Sidak's, Dunnett's, and Bonferroni's tests) conducted with Prism version 6.0 (GraphPad Software). All data are shown as the mean ± SEM. Statistical significance was set at *p* < 0.05. Statistical outliers twofold higher than SD were removed from analysis of *Nr4a1* mRNA in an MDD cohort. No data from animal studies were removed from analyses. For *in vivo* experiments, the number of dendritic spines was averaged per animal and per group (e.g., one to two dendritic segments/neuron, five to seven neurons/mouse; *N* = 5–7 mice as per group conditions). For *in vitro* experiments, the number of culture dishes per group was equivalent to the number of independent experiments. Estimates of sample size were calculated by power analysis based on preliminary data. Sample size was chosen to ensure 80% power to detect the prespecified effect size. Animals and culture dishes were attributed to various experimental groups in a random fashion. All data collected in animals were from littermate controls and were averaged per experimental groups. Knock-out animals were bred as heterozygotes to generate homozygote littermates. All data collected *in vitro* were replicates from independent experiments and averaged per experimental groups. Pre-established criteria for stopping data collection included the following: (1) mice electroporated in the wrong cortical regions; (2) mice reaching ethical end point limits; (3) unexpected mortality (e.g., one mouse NR4A1 KO injected with CORT died); and (4) brains badly perfused and unusable for histological studies.

## Results

### NR4A1 caused dendritic spine attrition *in vivo* via a genomic mechanism

To test whether NR4A1 regulates dendritic spine number postdevelopment *in vivo* when it is most expressed (Fig. 1-1; Chan et al., 1993; Davis and Puhl, 2011), we used a doxycycline-inducible construct to achieve temporal control of NR4A1 expression between P21 and P31 (Fig. 1*A*). Analysis of the apical tuft dendrites in NR4A1-overexpressing pyramidal neurons in PFC and S1 at P31 indicated that spine number was reduced upon exposure to doxycycline (two-way ANOVA for effect of doxycycline: *F*_(2,24)_ = 31.12, *p* < 0.0001, *post hoc* Bonferroni's test comparing 0 d with 3d, *p* = 0.0168; and comparing 0 d with 10 d, *p* < 0.0001; Fig. 1*B*). This was not an artifact of doxycycline given that neurons expressing GFP alone maintained similar spine density in the presence or absence of treatment (unpaired *t* test, *t*_(9)_ = 0.66, *p* = 0.52; Fig. 1*C*). These spines contain PSD-95 puncta juxtaposed with synaptophysin puncta suggestive of functional connectivity (Fig. 1-2).

10.1523/JNEUROSCI.2793-17.2017.f1-1 Figure 1-1 Developmental expression of NR4A1 in PFC Western blot with pan-NR4A1 antibody and GAPDH as loading control. Mean ±SE of N = 3 mice at each time point. Unpaired t-test for comparing the indicated time point with E18 *p < 0.035. Refers to Figure 1A. Download Figure 1-1, TIF file

10.1523/JNEUROSCI.2793-17.2017.f1-2 Figure 1-2 Synaptic markers of excitatory synapses in layer 2/3 pyramidal neurons of PFC. Confocal images of dendritic segments of pyramidal L2/3 neurons in PFC at PDN31 labelled with GFP plasmid electroporated in utero at E15.5, and co-stained with PDS95 and synaptophysin antibodies. Markers are where expected: PDS95 occurred in dendritic spines as puncta and dendritic spines were juxtaposed or near to synaptophysin puncta. Refers to Figure 1B. Download Figure 1-2, TIF file

**Figure 3.**
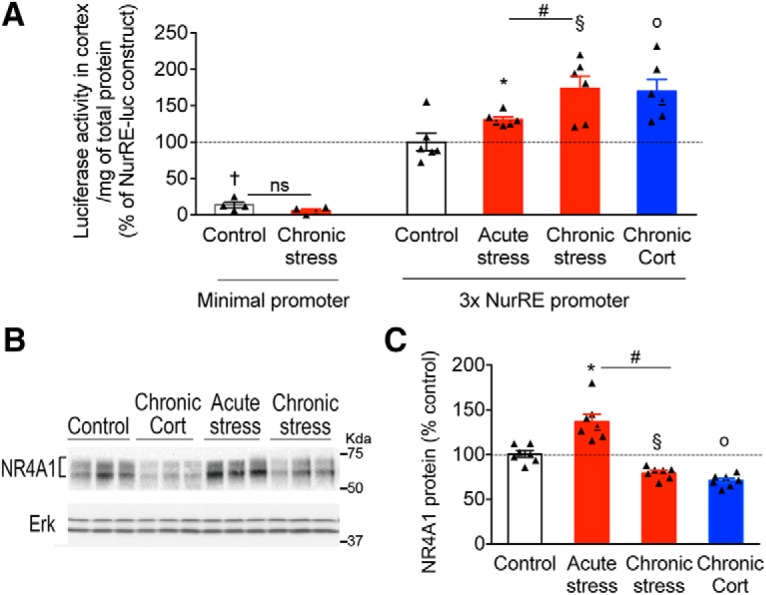
Chronic activation of the glucocorticoid stress pathway increased NR4A1 transcriptional activity in pyramidal L2/3 neurons of mouse PFC. ***A***, Luciferase activity at NR4A1 reporter NurRE-Luc compared with a minimal reporter construct introduced with a GFP reporter in cortical L2/3 neurons by electroporation *in utero*. Luc activity monitored at P31 *ex vivo* after microdissection of GFP-labeled PFC tissue. Data normalized to total milligrams of protein of GFP dissected tissues are expressed as a percentage of the NurRE-Luc construct in control resting mice (mean ± SEM of *N* = 4 mice with minimal promoter/group; *N* = 6 mice with NurRE/group, unpaired *t* test; †*p* = 0.0005, *p = 0.033, o*p* = 0.006, §*p* = 0.005 compared with controls; #*p* = 0.033 comparing acute and chronic stress). ***B***, Western blots of PFC lysates from P31 mice subjected to acute stress, chronic stress, or CORT injections compared with controls. ***C***, Protein levels of NR4A1 (mean ± SEM of *N* = 7 mice/group) expressed as a percentage of controls. Unpaired *t* test: *p = 0.0016, o*p* < 0.0001, §*p* < 0.0001 comparing indicated treatment to controls; #*p* < 0.0001 comparing acute and chronic stress.

10.1523/JNEUROSCI.2793-17.2017.f1-3 Figure 1-3 Cumulative distribution of spines density at apical tuft dendrites of L2/3 pyramidal neurons *in vivo*. (A) Comparisons with the Kolmogorov-Smirnov test between sc and #1 *p < 0.0001; sc and sc+stress *p < 0.0001; #1 and #1+stress p = 0.56; sc+stress and #1+stress §p < 0.0001 (Number of dendrites = 36 shRNA sc, 30 shRNA#1, 30 shRNA sc+stress and 23 shRNA#1+stress). Refers to Figure 1E. (B) Comparisons with the Kolmogorov-Smirnov test between sc and #1 *p = 0.0008; sc and sc+CORT *p < 0.0001; #1 and #1+CORT p = 0.1; sc+CORT and #1+CORT §p < 0.0001 (Number of dendrites = 34 shRNA sc+vehicle, 24 shRNA#1+vehicle, 30 shRNA sc+CORT and 27 shRNA#1+CORT). Refers to Figure 1F. Download Figure 1-3, TIF file

To determine whether NR4A1 used a genomic mechanism to reduce dendritic spine number *in vivo*, we electroporated doxycycline-inducible NR4A1 mutants: (1) ΔAF1 lacks the transactivation domain (Calnan et al., 1995); and (2) R337A cannot bind to DNA (Meinke and Sigler, 1999). Both mutants induced from P21 to P31 failed to diminish dendritic spine coverage in pyramidal neurons contrary to the wild-type (WT; main effect of NR4A1 constructs by two-way ANOVA: *F*_(3,36)_ = 9.82, *p* < 0.0001; effect of interaction of doxycycline with NR4A1 constructs: *F*_(3,36)_ = 5.95, *p* = 0.0021; *post hoc* Bonferroni's test for the effect of WT, *p* = 0.0031; ΔAF1, *p* = 0.12; and R337A, *p* = 0.97; Fig. 1*C*). These data indicated that NR4A1 required its transactivation domain for dendritic spine attrition *in vivo*.

### Stress- and CORT-mediated dendritic spine loss in cortex required NR4A1

NR4A1 expression in the adult brain is very sensitive to stress and CORT (Helbling et al., 2014), implying that it could contribute to dendritic spines attrition in such contexts. To address this possibility, we used ΔAF1 as a dominant-negative approach to block dendritic spine loss caused by chronic unpredictable and uncontrollable stress (main effect of stress compared with controls: two-way ANOVA, *F*_(1,19)_ = 39.89, *p* < 0.0001; *post hoc* Bonferroni's test, *p* = 0.0015; Fig. 1*C*). Induction of ΔAF1 in mouse cortex with doxycycline supplied during the entire stress paradigm from P21 to P31 preserved dendritic spines compared with induction of GFP (main effect of ΔAF1 by two-way ANOVA: *F*_(1,22)_ = 7.1, *p* = 0.0014; interaction of doxycycline with ΔAF1: *F*_(1,22)_ = 9.426, *p* = 0.0056; *post hoc* Bonferroni's test for the effect of ΔAF1: *p* = 0.0018; and GFP: *p* > 0.99; Fig. 1*C*). To confirm this result, we aimed at silencing the endogenous NR4A1 expression with shRNA sequences that are efficient at downregulating recombinant NR4A1 *in vitro* (Fig. 1*D*). The most efficient short hairpin (sh) sequence, sh#1, was introduced in cortex by *in utero* electroporation during embryogenesis and compared with its scrambled (sc) sequence as a control. Sh#1 prevented stress-induced spine loss (main effect of sh#1 by two-way ANOVA: *F*_(1,21)_ = 52.3, *p* < 0.0001; main effect of stress: *F*_(1,21)_ = 14.53, *p* = 0.001; interaction of sh#1 with stress: *F*_(1,21)_ = 4.72, *p* = 0.04; *post hoc* Bonferroni's test on scramble group: *p* = 0.0018; and *post hoc* Bonferroni's test on sh#1 group: *p* > 0.99; Fig. 1*E*), uniformly at the apical tuft dendrites (Fig. 1-3*A*). Sh#1 also prevented CORT-mediated spine loss (main effect of sh#1 by two-way ANOVA: *F*_(1,21)_ = 36.02, *p* < 0.0001; main effect of CORT: *F*_(1,21)_ = 13.15, *p* = 0.0016; interaction of sh#1 and CORT: *F*_(1,21)_ = 2.3; *post hoc* Bonferroni's test effect on scramble group: *p* = 0.0017; and *post hoc* Bonferroni's test effect on sh#1 group: *p* = 0.4; Fig. 1*F*), uniformly at the apical tuft dendrites (Fig. 1-3*B*). These results indicate that dendritic spine plasticity to stress and CORT depended on NR4A1 expression.

### Knockout of NR4A1 protected against adverse effects of chronic CORT exposure

We sought confirmation in NR4A1 knock-out mice using the chronic CORT exposure model. Morphological analysis of Golgi–Cox-impregnated pyramidal neurons (Fig. 2*A*) showed that the distribution of dendritic spines (Fig. 2*B*) and the overall density in PFC was denser in −/− than in +/+ mice after chronic CORT exposure (main effect of genotype by two-way ANOVA: *F*_(1,16)_ = 26.37, *p* < 0.0001; main effect of CORT: *F*_(1,16)_ = 105.6, *p* < 0.0001; interaction of genotype and CORT: *F*_(1,16)_ = 19.2, *p* = 0.0005; *post hoc* Bonferroni's test: *p* < 0.0001; and effect of CORT injection: *F*_(1,16)_ = 105.6; *post hoc* Bonferroni's test: *p* < 0.0001; Fig. 2*C*). Another typical adverse effect of chronic CORT exposure is behavioral despair in the TST (Nestler and Hyman, 2010) in which −/− mice performed better than +/+ mice (main effect of genotype by two-way ANOVA: *F*_(1,91)_ = 26.34, *p* < 0.0001; *post hoc* Sidak's *t* test comparing total time immobile between genotypes: *p* < 0.05; Fig. 2*B*). These data indicated that the knockout of NR4A1 protected against some adverse effects of chronic CORT exposure.

### Chronic stress and CORT exposure increased NR4A1 activity in mouse cortex

Knockdown, knockout, and dominant-negative data all suggested that NR4A1 could be more active upon stimulation of the glucocorticoid stress pathway than at rest. To monitor NR4A1 activity in pyramidal neurons of PFC, we established a transcription-based Luciferase assay *in vivo*. A synthetic promoter harboring 3× tandem NR4A1-reponsive elements (NurREs) upstream of Luciferase showed endogenous activity in mouse cortex at rest at P31 compared with cortical tissue electroporated with the minimal promoter lacking NurRE (unpaired *t* test: *t*_(8)_ = 5.63, *p* = 0.0005; Fig. 3*A*). Using this assay, we found that chronic CORT exposure and stress increased the activity of neuronal NR4A1 in mouse cortex compared with controls at rest (acute stress: unpaired *t* test, *t*_(10)_ = 2.46, *p* = 0.033; chronic stress: unpaired *t* test, *t*_(10)_ = 3.53, *p* = 0.005; and chronic CORT administration: unpaired *t* test, *t*_(10)_ = 3.46, *p* = 0.006). Remarkably, chronic stress had a stronger effect than acute stress on activating NR4A1 in PFC (unpaired *t* test: *t*_(10)_ = 2.45, *p* = 0.033).

To confirm this result, we used Western blot to detect NR4A1 total protein levels in PFC lysates (Fig. 3*B*,*C*). Acute stress increased NR4A1 levels compared with controls (unpaired *t* test: *t*_(12)_ = 4.06, *p* = 0.0016), whereas chronic stress decreased NR4A1 levels compared with controls (unpaired *t* test: *t*_(12)_ = 4.72, *p* = 0.005). We found the same comparing chronic CORT to control groups (unpaired *t* test: *t*_(12)_ = 6.65, *p* < 0.001). The difference between acute and chronic stress was significant (unpaired *t* test: *t*_(12)_ = 6.72, *p* < 0.0001; Fig. 3*C*), featuring a possible sensitization phenomenon. One issue raised by these data is the discrepancy between NR4A1 activity and NR4A1 levels in the context of chronic stress and CORT exposure.

### Transcriptional activity of neuronal NR4A1 depended on its nuclear localization, phosphorylation, and turnover

**Figure 4.**
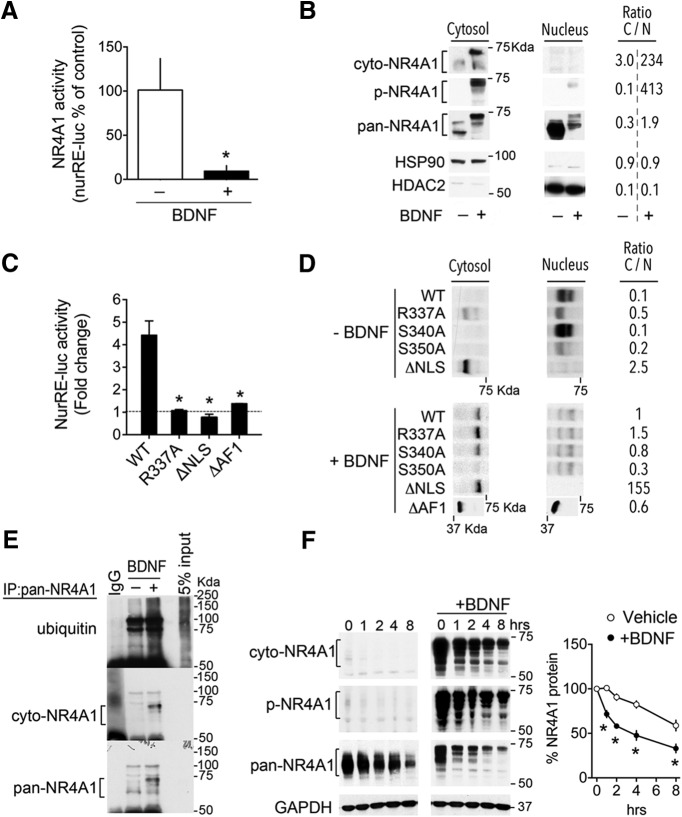
NR4A1 transcriptional activity in cortical neurons depends on its nuclear localization, phosphorylation, and turnover. ***A***, NR4A1 activity expressed as a percentage of untreated controls impeded after stimulation with 25 ng/ml BDNF for 3 h (mean ± SEM of four independent experiments; unpaired *t* test, *p = 0.042). NurRE-Luc reporter was cotransfected with recombinant NR4A1 in primary cortical neurons by electroporation and Luciferase activity monitored in lysates. ***B***, Antibodies to detect the nucleo-cytoplasmic isoforms (pan-NR4A1), cytosolic isoform (cyto-NR4A1), and phosphorylated isoforms (p-NR4A1 at Ser350) of recombinant NR4A1 electroporated in primary cortical neurons. Ratio of proteins in cytosol (C) vs nuclear (N; means of four independent samples/group) altered after stimulation with 25 ng/ml BDNF for 3 h. HSP90 and HDAC2 are loading controls. ***C***, NurRE-Luc reporter activity in primary cortical neurons transfected with the indicated constructs. Data normalized to GFP controls (mean ± SEM of three independent experiments; ANOVA *post hoc* Dunnett's test comparing WT and mutants, *p < 0.0005). ***D***, Deletion of Ser350 or NLS sequences altered NR4A1 C/N ratio in primary cortical neurons (means of four independent samples/group normalized to WT+BDNF). ***E***, Polyubiquitination of NR4A1 immunoprecipitated from lysates of primary cortical neurons stimulated with 25 ng/ml BDNF for 3 h. ***F***, Withdrawal of doxycycline for the indicated time destabilized NR4A1 recombinant protein levels in primary cortical neurons. Mean ± SEM of four independent experiments; ANOVA *post hoc* Sidak's test for interaction of BDNF and time, *p < 0.0001.

We explored the possibility that the activity of neuronal NR4A1 could depend on nuclear export besides its levels. For this, we used BDNF as a tool in an *in vitro* system to force NR4A1 nuclear export in cortical neurons because TrkB is abundant in pyramidal cortical neurons (Jeanneteau et al., 2008), NR4A1 nuclear export was achieved in PC12 cells with a related neurotrophin (Katagiri et al., 2000), and BDNF expression is decreased by chronic stress and chronic CORT exposure (Duman, 2004; Arango-Lievano et al., 2015), which is relevant to the neuropathology of stress-related disorders (Autry and Monteggia, 2012). As anticipated, BDNF suppressed the transcriptional activity of NR4A1 in primary cortical neurons (unpaired *t* test: *t*_(6)_ = 2.57, *p* = 0.042; Fig. 4*A*). Subcellular fractionation to separate nuclear proteins from the cytosol indicated that NR4A1 was mostly nuclear in untreated neurons and mostly cytosolic after BDNF treatment (Fig. 4*B*). Furthermore, we characterized the following three antibodies to distinguish between the cytosolic and nuclear isoforms of NR4A1 (Fig. 4*B*): (1) the antibody against the phosphorylated residue Ser350 (p-NR4A1); (2) the antibody raised against the epitope-containing residues 325–374 (cyto-NR4A1); and (3) the antibody against both nuclear and cytosolic isoforms for total protein levels (pan-NR4A1).

To demonstrate the link among NR4A1 transcriptional activity, phosphorylation, and nuclear export, we used NR4A1 mutants. Deletion of the phosphorylation site (S350A), the ΔNLSs, the DNA-binding motif (R337A), or the transactivation domain (ΔAF1), all impaired NR4A1 nucleo-cytoplasmic ratio and transcriptional activity in primary cortical neurons (main effect of mutants by ANOVA: *F*_(3,8)_ = 28, *p* = 0.0001; *post hoc* Dunnett's test for comparing ΔNLS and WT: *p* = 0.0001; *post hoc* Dunnett's test for comparing R337A and WT: *p* = 0.0002; *post hoc* Dunnett's test for comparing ΔAF1 and WT: *p* = 0.0004; Fig. 4*C*,*D*). Additionally, NR4A1 ubiquitination was higher at the cytosolic isoform than the nuclear isoform (Fig. 4*E*), and NR4A1 downregulation was higher at the cytosolic isoform than the nuclear isoform (main effect of BDNF by two-way ANOVA: *F*_(1,6)_ = 38.24, *p* = 0.0008; interaction of BDNF with time: *F*_(4,24)_ = 12.18, *p* < 0.0001; *post hoc* Sidak's test for comparing BDNF and vehicle: at 2, 4, and 6 h, *p* < 0.0001; at 8 h, *p* = 0.0002; Fig. 4*F*). Altogether, these data indicated that cytosolic NR4A1 was not transcriptionally active and most likely was degraded.

### Stress and CORT altered the nuclear localization, phosphorylation, and levels of NR4A1

**Figure 5.**
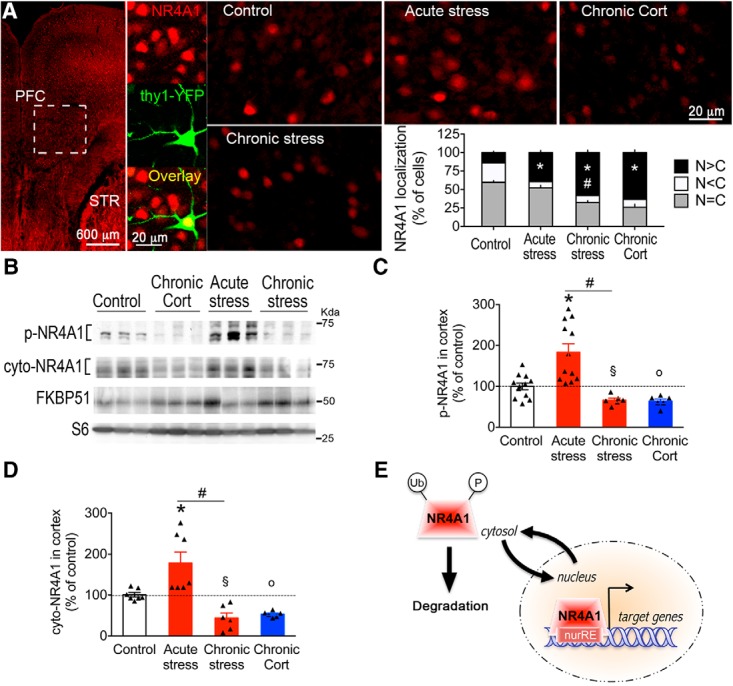
Chronic activation of the glucocorticoid stress pathway decreased NR4A1 phosphorylation and cytosolic distribution in mouse PFC. ***A***, Endogenous NR4A1 protein is both nuclear and cytosolic in pyramidal cortical neurons of PFC (*thy1-*YFP). Proportion of a total of 10,600 cells with NR4A1 localized equally in nucleus and cytosol (N = C), more in nucleus than cytosol (N > C), and less in nucleus than cytosol (N < C). Mean ± SEM of *N* = 10 controls, *N* = 10 acute stress mice, *N* = 7 CORT mice, and *N* = 9 chronic stress mice; unpaired *t* test: *p = < 0.0001 comparing indicated group to controls; and #*p* = 0.0006 comparing acute to chronic stress mice. ***B***, Western blots of PFC lysates from P31 mice subjected to acute stress, chronic stress, or CORT injections compared with controls. ***C***, Phosphorylation of NR4A1 (S350-P) expressed as a percentage of controls. Mean ± SEM of *N* = 12 control mice, *N* = 12 acute stress mice, *N* = 6 CORT mice, and *N* = 6 chronic stress mice; unpaired *t* test: *p = 0.013, o*p* = 0.0094, §*p* = 0.014 comparing indicated treatment to controls; #*p* = 0.0153 comparing acute and chronic stress mice. ***D***, Cytosolic NR4A1 expressed as a percentage of controls. Mean ± SEM of *N* = 7 control mice, *N* = 7 acute stress mice, *N* = 6 CORT mice, and *N* = 6 chronic stress mice; unpaired *t* test: *p = 0.025, o*p* < 0.0001, §*p* = 0.0009 comparing indicated treatment to controls; #*p* = 0.0025 comparing acute and chronic stress mice. ***E***, Model of NR4A1 functional transport in cortical neurons. Induction of NR4A1 by acute stress increased nuclear export and phosphorylation. Chronic stress prolonged NR4A1 nuclear residency, which failed to contain its activity.

To determine NR4A1 subcellular localization *in vivo*, we used the antibodies characterized *in vitro*. The pan-NR4A1 antibody detected total levels in both nucleus and cytosol of pyramidal neurons (labeled with *thy1*-YFP) in mouse PFC at rest (Fig. 5*A*). The nucleus/cytosol ratio of pan-NR4A1 increased by acute stress (unpaired *t* test: *t*_(18)_ = 7.53, *p* < 0.0001), chronic stress (unpaired *t* test: *t*_(17)_ = 12.2, *p* < 0.0001) and chronic CORT exposure (unpaired *t* test: *t*_(15)_ = 14.1, *p* < 0.0001). We used Western blot to quantify p-NR4A1 and cyto-NR4A1 in PFC lysates, as the antibodies did not work for histology (Fig. 5*B*). Acute stress increased p-NR4A1 compared with controls (unpaired *t* test: *t*_(22)_ = 2.69, *p* = 0.013; Fig. 5*C*) and also increased cyto-NR4A1 compared with controls (unpaired *t* test: *t*_(12)_ = 2.55, *p* = 0.025; Fig. 5*D*). In contrast, we found the opposite comparing chronic stress to controls [effect on p-NR4A1: unpaired *t* test, *t*_(16)_ = 2.74, *p* = 0.014 (Fig. 5*C*); effect on cyto-NR4A1 levels: unpaired *t* test: *t*_(11)_ = 4.48, *p* = 0.0009 (Fig. 5*D*)] and chronic CORT exposure to controls [effect on p-NR4A1: unpaired *t* test, *t*_(16)_ = 2.95, *p* < 0.009 (Fig. 5*C*); effect on cyto-NR4A1 levels: unpaired *t* test, *t*_(12)_ = 7.53, *p* < 0.0001 (Fig. 5*D*)]. This was not a generalized effect on proteins sensitive to stress and CORT exposure because FKBP51 was induced in these contexts (Fig. 5*B*).

Again, different effects between acute and chronic stress on pan-NR4A1 nucleus/cytosol ratio (unpaired *t* test: *t*_(17)_ = 4.18, *p* = 0.0006; Fig. 5*A*), p-NR4A1 (unpaired *t* test: *t*_(16)_ = 2.71, *p* = 0.015; Fig. 5*C*) and cyto-NR4A1 (unpaired *t* test: *t*_(11)_ = 3.9, *p* = 0.0025; Fig. 5*D*), featured a putative sensitization phenomenon. Based on *in vitro* and *in vivo* data, we proposed a hypothetical model (Fig. 5*E*) in which stress signals could influence transcriptional activity of neuronal NR4A1 by phosphorylation, nuclear export, and degradation by the ubiquitin–proteasome system.

### Genes regulated by neuronal NR4A1 are involved in mitochondrial uncoupling

NR4A1 activity regulates the expression of genes involved in cellular metabolism and cytoskeleton structure (Chao et al., 2007; Fassett et al., 2012; Chen et al., 2014). Here, we examined the expression of mitochondria-regulatory genes because both neuronal mitochondria and neuronal excitability were influenced by NR4A1 in a seizure model (Zhang et al., 2009). Compared with GFP, NR4A1 modified the expression of uncoupling proteins (*Ucp2:* unpaired *t* test, *t*_(6)_ = 5.1, *p* = 0.002; *Ucp4*: unpaired *t* test, *t*_(6)_ = 2.18, *p* = 0.035; Table 1), an effect blocked by BDNF as it caused NR4A1 nuclear export in cortical neurons. We used glutamate rather than CORT to simulate the effects of chronic stress *in vitro* because inhibitors of glutamate receptors blocked the effects of CORT and chronic stress on dendritic spine plasticity and neurotransmission (Duman, 2004; Popoli et al., 2011). We found that NR4A1 regulated more genes in the context of glutamate stimulation, as follows: *Ucp4* (unpaired *t* test: *t*_(6)_ = 4.94, *p* = 0.0026); *Mfn1* (unpaired *t* test: *t*_(6)_ = 2.03, *p* = 0.044); *Fis1* (unpaired *t* test: *t*_(6)_ = 3.48, *p* = 0.013); and *Opa1* (unpaired *t* test: *t*_(6)_ = 2.3, *p* = 0.033), unraveling a context-dependent effect. The consistent upregulation of *Ucp4* mRNA at rest and after glutamate stimulation suggested that NR4A1 could affect ATP stocks by uncoupling mitochondria proton transport from respiration (Liu et al., 2006; Klotzsch et al., 2015).

**Table 1.** Effect of neuronal NR4A1 on mitochondria-regulatory gene expression

	Treatments															
	*DRP1*		*MFN1*		*MFN2*		*FIS1*		*OPA1*		*UCP2*		*UCP4*		*UCP5*	
	%	SE	%	SE	%	SE	%	SE	%	SE	%	SE	%	SE	%	SE
Ratio (NR4A1/GFP)																
Untreated controls	127.6	15.3	128.7	28.4	113.5	13.4	106.6	6.8	111.0	8.4	22.3**	14.9	157.5**	26.3	112.8	17.8
25 ng/ml BDNF	119.5	16.9	132.3	22.0	104.8	11.9	97.9	8.8	75.7	8.3	87.4	10.6	126.4	16.2	77.8	11.8
25 mm glutamate	95.9	7.9	127.9	10.2	96.7	20.1	129.6	8.2	107.8	11.0	85.6	17.4	157.9	30.5	88.9	14.5
100 mm glutamate	121.3	16.6	70.1*	3.5	90.1	4.5	159.8*	20.7	155.5*	24.7	61.5	28.6	176.9*	14.9	98.2	9.2

Messenger RNA levels determined by qPCR on total RNA extracts of primary cortical neurons (DIV14 with 10 d of doxycycline treatment). Data are expressed as a percentage of GFP controls (mean ± SE of *N* = 4 independent experiments). Unpaired *t* test for comparing the effect of NR4A1 with GFP in untreated control cells.

**p < 0.036, for comparing effect of NR4A1 with GFP in BDNF-treated cells (25 ng/ml for 3 h).

*p < 0.045 for comparing the effect of NR4A1 with GFP in glutamate-treated cells (100 μm for 3 h).

### NR4A1 transactivation domain was required to elicit mitochondrial proton leak

**Figure 6.**
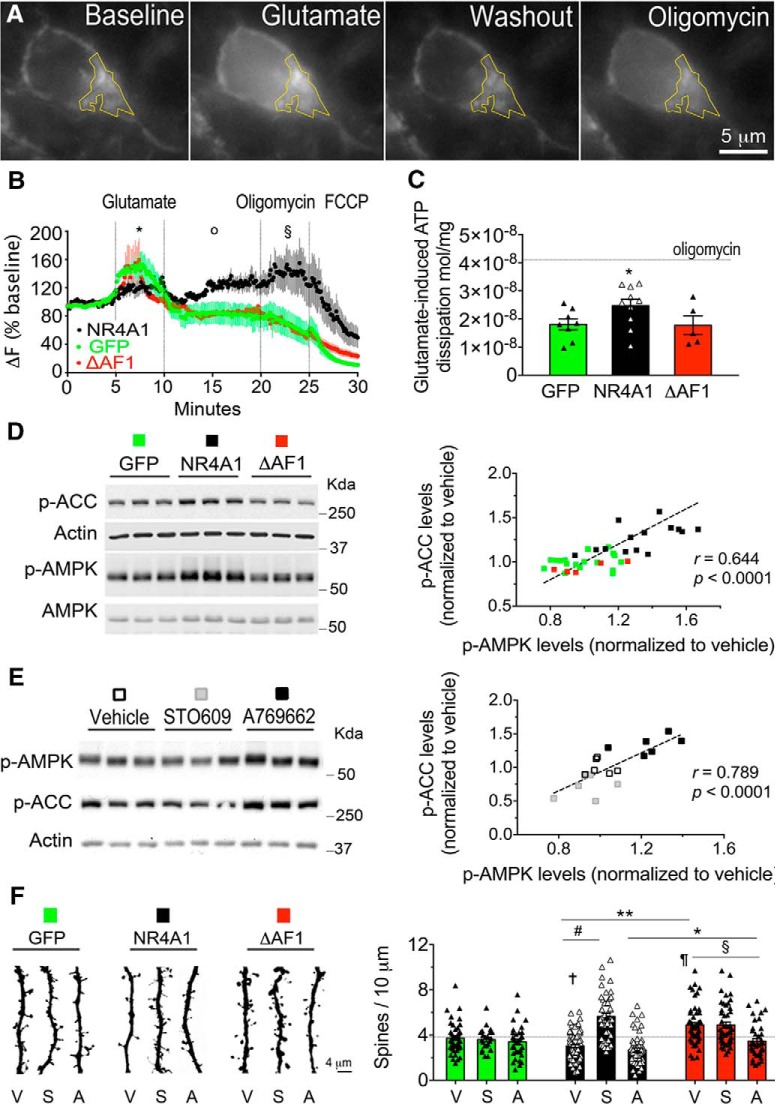
Activation of the NR4A1–AMPK pathway reduced dendritic spine number *in vitro*. ***A***, Time-lapse imaging of TMRM fluorescence in primary cortical neurons (DIV14 with 10 d of doxycycline treatment). Yellow box is one example of an ROI. ***B***, Δ*F* (*F* − *F*_0_/*F*_0_ × 100) traces are the mean ± SEM of *N* = 10 GFP, *N* = 10 NR4A1, and *N* = 7 ΔAF1 neurons subjected to all treatments subsequently. Statistical comparison between GFP and NR4A1 groups at indicated epochs (last 3 min of each treatment) by unpaired *t* test: during 100 μm glutamate, *p = 0.009 (*N* = 16 GFP, *N* = 27 NR4A1 cells); during washout, o*p* = 0.0039 (*N* = 16 GFP, *N* = 27 NR4A1 cells); and during 1 μm oligomycin administration, §*p* = 0.035 (*N* = 10 GFP, *N* = 10 NR4A1 cells). ***C***, Dissipation of ATP upon stimulation of primary cortical neurons (DIV14 with 10 d of doxycycline treatment) with 100 μm glutamate compared with 1 μm oligomycin for 1 h. Mean ± SEM of *N* = 8 GFP, *N* = 10 NR4A1, and *N* = 5 ΔAF1 independent samples; unpaired *t* test comparing GFP and NR4A1, *p = 0.024. ***D***, Phosphorylation of AMPK and its substrate ACC in primary cortical neurons (DIV14 with 10 d of doxycycline treatment) after stimulation with 25 μm glutamate for 3 h (*N* = 18 GFP, *N* = 15 NR4A1, *N* = 5 ΔAF1 samples/group). Pearson correlation between levels of p-ACC and p-AMPK normalized to GFP as controls. Group comparison by unpaired *t* test for p-ACC: NR4A1 vs GFP, *p* < 0.0001; and ΔAF1 vs GFP, *p* = 0.009; for p-AMPK: NR4A1 vs GFP, *p* < 0.0001; and ΔAF1 vs GFP, *p* = 0.004. ***E***, Modulation of AMPK by 10 μm STO609 and 1 μm A769662 for 24 h in primary cortical neurons (DIV14). Pearson correlation between levels of p-ACC and p-AMPK normalized to vehicle controls (*N* = 6 samples/group). Comparison by unpaired *t* test for p-ACC: STO609 vs vehicle, *p* = 0.0061; and A769662 vs vehicle, *p* = 0.0007; for p-AMPK: STO609 vs vehicle, *p* = 0.2; and A769662 vs vehicle; *p* = 0.0013. ***F***, Effect of STO609 (S) and A769662 (A) compared with vehicle (V) on dendritic spine density in primary cortical neurons expressing GFP, NR4A1, or ΔAF1 (DIV21 with 10 d of doxycycline treatment). Mean ± SEM of *N* = 99 GFP, *N* = 192 NR4A1, and *N* = 160 ΔAF1 dendrites; ANOVA *post hoc* Tukey's test for comparing the following: NR4A1 and ΔAF1, **p < 0.0001; GFP and NR4A1, †*p* = 0.0019; GFP and ΔAF1, ¶*p* = 0.002; STO609 and vehicle on NR4A1 cells, #*p* < 0.0001; A769662 and vehicle on ΔAF1 cells, §*p* = 0.023; interaction of NR4A1 and A769662, *p < 0.045. See extended data Figure 6-1.

To monitor mitochondrial membrane potential that depends on the mitochondrial proton gradient, we used time-lapse imaging of the potentiometric mitochondrial dye TMRM in neurons (Fig. 6*A*). Compared with GFP, NR4A1 reduced the following: (1) mitochondrial membrane depolarization to glutamate (unpaired *t* test: *t*_(41)_ = 2.73, *p* = 0.0091); (2) mitochondria membrane repolarization during washout (unpaired *t* test: *t*_(41)_ = 3.05, *p* = 0.0039); and (3) mitochondria energetic competence in the oligomycin test (unpaired *t* test: *t*_(18)_ = 2.27, *p* = 0.035) because it hyperpolarized mitochondria in GFP neurons but depolarized them in NR4A1-transfected neurons (Ward et al., 2000). These observations (Fig. 6*B*) indicated that NR4A1, unlike its mutant ΔAF1, caused mitochondrial uncoupling.

10.1523/JNEUROSCI.2793-17.2017.f6-1 Figure 6-1 Cumulative distribution of dendritic spines density in cultured cortical neurons. Comparisons with the Kolmogorov-Smirnov test between GFP and NR4A1 *p = 0.005; GFP and ΔAF1 *p = 0.001; NR4A1 and ΔAF1 *p < 0.0001; for the effect of STO609 between GFP and NR4A1 #p < 0.0001; GFP and ΔAF1 #p = 0.036; NR4A1 and ΔAF1 #p = 0.0016; for the effect of A769662 between GFP and NR4A1 op = 0.001; NR4A1 and ΔAF1 op = 0.031 (Number of dendrites = 99 GFP (V: 42; S: 23; A: 34), 192 NR4A1 (V: 85; S: 47; A: 60) and 160 ΔAF1 (V: 57; S: 47; A: 56). Refers to Figure 6F. Download Figure 6-1, TIF file

Stimulation with glutamate decreased whole-cell ATP levels (main effect of glutamate: −59 ± 2.9%; two-way ANOVA: *F*_(1,10)_ = 106.8, *p* < 0.0001; *post hoc* Bonferroni's test: *p* = 0.0007). This use of ATP stocks was exaggerated by NR4A1 compared with GFP (unpaired *t* test: *t*_(16)_ = 2.14, *p* = 0.024; Fig. 6*B*) but was far from the ATP-depleting effect of oligomycin (unpaired *t* test: *t*_(10)_ = 3.17, *p* = 0.009; Fig. 6*C*). Consistent with a mild shortage of ATP, phosphorylation of the AMPK increased modestly by NR4A1 compared with GFP (+33.8 ± 5.2%; unpaired *t* test: *t*_(31)_ = 5.55, *p* < 0.0001; Fig. 6*D*) and phosphorylation of its substrate ACC increased modestly by NR4A1 compared with GFP (+34 ± 7.8%; unpaired *t* test: *t*_(31)_ = 4.54, *p* < 0.0001; Fig. 6*D*). In contrast, ΔAF1 lacking transcriptional activity had no effects on AMPK signaling.

### NR4A1 used the AMPK catabolic pathway to reduce dendritic spine number

To clarify a hypothetical link between AMPK signaling and morphological plasticity evoked by NR4A1, we used pharmacological modulators (Fig. 6*E*). We obtained mild modulation of AMPK in primary cortical neurons by STO609 (−7 ± 4.1% for p-AMPK and −29 ± 6.8% for p-ACC; unpaired *t* test: *t*_(10)_ = 3.46, *p* = 0.006) and by A769662 (p-AMPK: +24.2 ± 4.9%; unpaired *t* test, *t*_(10)_ = 4.43, *p* = 0.0013; p-ACC: +33.8 ± 5.3%, unpaired *t* test, *t*_(10)_ = 4.79, *p* = 0.0007), respectively, inhibitor of calcium/calmodulin-dependent protein kinase kinase (CAMKK) activating kinase of AMPK (Mairet-Coello et al., 2013) and activator of AMPK (Scott et al., 2014). In transfected neurons, we found a functional interaction between NR4A1 and STO609 that reverted the low dendritic spine number above control levels (main effect of constructs by two-way ANOVA: *F*_(2,296)_ = 12.06, *p* < 0.0001; main effect of STO609: *F*_(1,296)_ = 16.5, *p* < 0.0001; interaction of constructs and STO609: *F*_(2,296)_ = 21.28, *p* < 0.0001; *post hoc* Tukey's test for comparing STO609 and vehicle: on NR4A1 cells, *p* < 0.0001; on ΔAF1 cells, *p* > 0.99; on GFP cells, *p* = 0.99; Fig. 6*F*; for cumulative distributions, see Fig. 6-1). Such an interaction was absent between ΔAF1 and STO609, suggesting redundancy between the NR4A1 and AMPK pathways.

We tested further this hypothetical framework with the AMPK activator A769662, as it is predicted to reduce dendritic spine number in neurons expressing ΔAF1 but not in neurons expressing NR4A1. We found no functional interaction between NR4A1 and A769662. On the contrary, A769662 reverted the effect of ΔAF1 on dendritic spine number (main effect of constructs by two-way ANOVA: *F*_(2,300)_ = 22.8, *p* < 0.0001; main effect of A769662: *F*_(2,300)_ = 14.03, *p* = 0.0002; interaction of constructs and A769662: *F*_(1,300)_ = 1.96, *p* = 0.14; *post hoc* Tukey's test for comparing A769662 and vehicle: on ΔAF1 cells, *p* = 0.0039; on NR4A1 cells, *p* = 0.6; on GFP cells, *p* = 0.79; Fig. 6*F*; for cumulative distributions, see Fig. 6-1).

**Figure 7.**
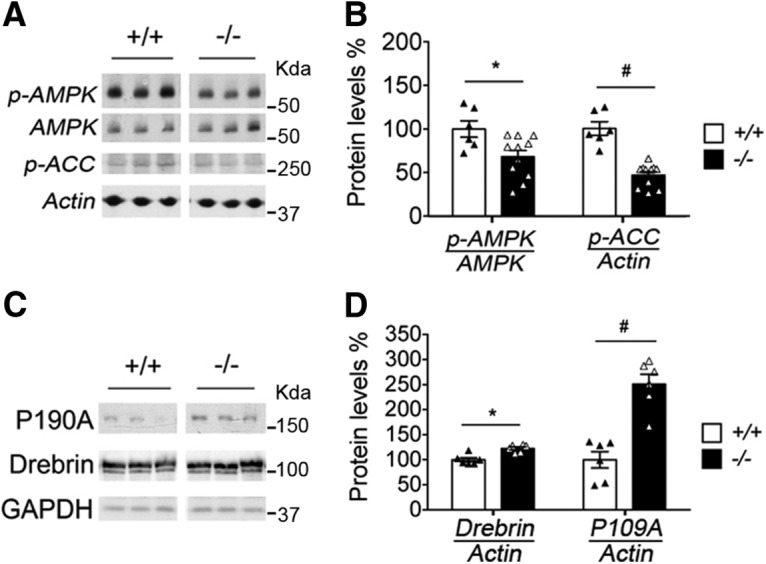
AMPK signaling and levels of synaptic markers in PFCs of NR4A1 knockouts. ***A***, AMPK signaling in PFCs of NR4A1 KO rats. ***B***, Mean ± SEM of 11 −/− mice compared with 6 +/+ mice. Data expressed as a percentage of controls (unpaired *t* test comparing genotypes: *p = 0.017 and #*p* = 0.007). ***C***, Levels of synaptic markers in PFCs of NR4A1 KO rats. ***D***, Mean ± SEM of 6 −/− mice compared with 6 +/+ mice. Data expressed as a percentage of controls (unpaired *t* test comparing genotypes: *p = 0.0013 and #*p* = 0.0002). See extended data Figure 7-1.

To confirm *in vivo* the functional link between AMPK and NR4A1 pathways, we used NR4A1 knockouts (Fig. 7). An effect of genotype on p-AMPK (unpaired *t* test: *t*_(15)_ = 2.66, *p* = 0.017) and p-ACC was observed in the PFC (unpaired *t* test: *t*_(15)_ = 6.8, *p* = 0.0074). An effect of genotype was also observed on the levels of protein markers of excitatory synapses (P190A: unpaired *t* test, *t*_(10)_ = 4.42, *p* = 0.0013; Drebrin: unpaired *t* test, *t*_(10)_ = 5.88, *p* = 0.0002; Fig. 7*D*). This is consistent with the decrease of protein markers of excitatory synapses in primary cortical neurons expressing NR4A1 compared with ΔAF1 (Fig. 7-1). Altogether, these data indicated that AMPK signaling is an effector pathway of NR4A1 that alters dendritic spine number.

10.1523/JNEUROSCI.2793-17.2017.f7-1 Figure 7-1 NR4A1 acts as transcription factor to decrease synaptic proteins content. Doxycycline-induced expression of NR4A1 decreased levels of excitatory synapse proteins in primary cortical neurons (DIV14, doxycycline treatment for last 10 days). This effect required NR4A1 transcriptional activity given that doxycycline-induced expression of ΔAF1 had no effect. Mean ±SEM of N = 4 independent experiments. Group comparisons between doxycycline and no doxycycline at the indicated markers for the effect of NR4A1 by unpaired t-test *p < 0.001, **p < 0.0005, for the effect of ΔAF1 #p < 0.014, and for comparing NR4A1 and ΔAF1 at the indicated markers =*p* = 0.03, ==*p* = 0.002, ¶*p* < 0.004. Refers to Figure 7D. Download Figure 7-1, TIF file

### NR4A1 levels in human PFC of MDD patients

**Figure 8.**
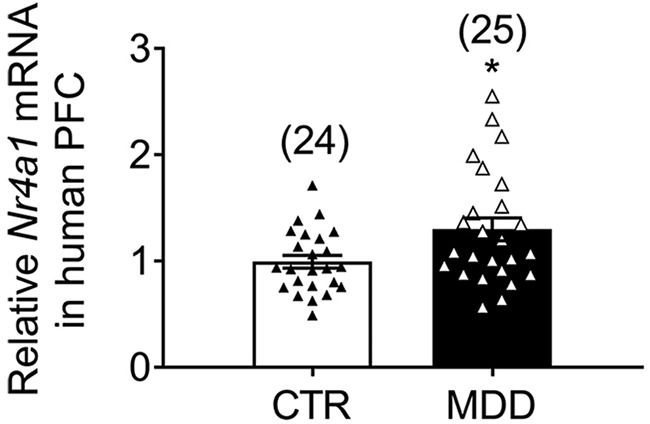
Abnormal NR4A1 transcript levels in human MDD brains. Upregulated levels of *NR4A1* mRNA in PFCs of MDD subjects. Mean ± SEM normalized to *Tubulin* (*N* = 25 CTR, *N* = 24 MDD; unpaired *t* test: *t*_(47)_ = 3.13, *p = 0.018). For demographic information, see extended data Figure 8-1.

Mechanistic data support a causative role of NR4A1 on synaptic loss in PFC neurons in the context of stress and disrupted CORT levels, as both increased its neuronal activity in animal models. In humans, synaptic loss in PFC neurons and disrupted CORT levels are established neuropathological features of MDDs that are consistent with a putative impaired activity of NR4A1 (Duman et al., 2016). In a previous study (Duric et al., 2010), we showed that *Nr4a1* transcripts were upregulated by 1.72-fold (*p* = 0.029, *t* test corrected for false discovery rate) in a whole-genome microarray profiling of human hippocampus in MDDs. In the same human cohort (for demographic information, see Fig. 8-1), we analyzed *Nr4a1* mRNA levels in the medial PFC. Quantitative PCR analysis showed that *Nr4a1* transcripts were significantly higher in PFC of MDD subjects (increase, 1.39-fold; unpaired *t* test: *t*_(47)_ = 3.13, *p* = 0.018) when compared with matched psychiatrically healthy control subjects (Fig. 8). Factorial analysis revealed no effect of sex (*p* = 0.372) and no effect of antidepressant medication prescription (*p* = 0.139).

10.1523/JNEUROSCI.2793-17.2017.f8-1 Figure 8-1 Demographic information of MDD subjects and controls. F, female; M, male; PFC, prefrontal cortex (medial: Brodmann area 9); ND, no psychotropic medication detected; PMI, postmortem interval (hours); 1Psychotrophic prescriptions within last month. Refers to Figure 8. Download Figure 8-1, TIF file

### NR4A1 levels in human PFC of AD patients

**Figure 9.**
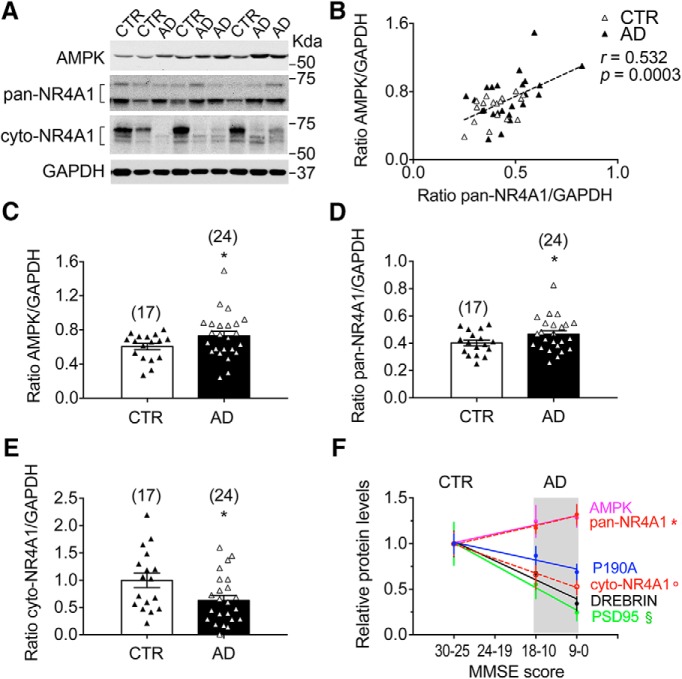
Abnormal levels of AMPK and NR4A1 in human AD brains. ***A***, Representative image of AMPK protein and NR4A1 nucleo-cytoplasmic isoforms in human cortex. See extended data Figure 9-1. ***B***, Pearson correlation (*p* = 0.0003) between AMPK and pan-NR4A1 levels in human PFCs from AD (*N* = 24) and CTR (*N* = 17). Data are Western blot optical densities normalized to GAPDH. ***C***, Upregulation of AMPK protein levels in human PFCs (mean ± SEM of *N* = 24 AD subjects and *N* = 17 control subjects (CTRs) normalized to GAPDH levels; unpaired *t* test, *p = 0.035). ***D***, Downregulation of NR4A1 cytoplasmic isoform in human PFCs (mean ± SEM of *N* = 24 AD subjects and *N* = 17 CTRs normalized to GAPDH levels; unpaired *t* test, *p = 0.024). ***E***, Upregulation of NR4A1 total isoforms in human PFCs (mean ± SEM of *N* = 24 AD subjects and *N* = 17 CTRs normalized to GAPDH levels; unpaired *t* test, *p = 0.0258). ***F***, Pearson correlations between MMSE scores and levels of AMPK (*p* = 0.011), cyto-NR4A1 (*p = 0.041), pan-NR4A1 (o*p* = 0.019), or synaptic markers PSD-95 (§*p* = 0.013), Drebrin (*p* = 0.003), and P190A (#*p* = 0.049). Data are normalized to CTRs (mean ± SEM of *N* = 17 subjects with a score of 30–25, *N* = 12 subjects with a score of 18–10, and *N* = 12 subjects with a score of 9–0). For details, see extended data Figure 9-1.

Similarities in AD and MDD with respect to volume reduction, neuronal atrophy, and loss of connectivity in PFC could underlie common deficits in intracellular signaling network (van Veluw et al., 2012; Sampath et al., 2017). Synaptic loss in PFC neurons and disrupted CORT levels are neuropathological features of cognitive deficits across disorders with overlap in the expression of clinical state, established in MDD and AD (Uylings and de Brabander, 2002; Knobloch and Mansuy, 2008; Lupien et al., 2009). This prompted us to assess NR4A1 levels in PFC of human subjects clinically diagnosed with AD and age-matched cognitive healthy control subjects (for demographic information, see Fig. 9-1). We used Western blots to discriminate between pan-NR4A1 and cyto-NR4A1 levels, as NR4A1 activity cannot be determined postmortem (Fig. 9*A*). Levels of pan-NR4A1 correlated with those of AMPK (Pearson *r* = 0.53, *p* = 0.0003; Fig. 9*B*). Higher levels of AMPK (unpaired *t* test: *t*_(39)_ = 2.17, *p* = 0.035; Fig. 9*C*) and pan-NR4A1 (unpaired *t* test: *t*_(39)_ = 2.31, *p* = 0.025; Fig. 9*D*) were found in AD patients compared with control subjects. But lower levels of cyto-NR4A1 were found in AD patients compared with control subjects (unpaired *t* test: *t*_(39)_ = 2.34, *p* = 0.024; Fig. 9*E*). These data provided a molecular signature (Fig. 9*F*) proportionate to cognitive scores on the MMSE test (for cyto-NR4A1: Pearson *r* = −0.37, *p* = 0.041; pan-NR4A1: Pearson *r* = 0.36, *p* = 0.019; AMPK: Pearson *r* = 0.48, *p* = 0.011; Fig. 9-1*B*) and to the levels of synaptic markers (PSD-95: Pearson *r* = −0.38, *p* = 0.013; Drebrin: Pearson *r* = −0.44, *p* = 0.003; P190A: Pearson *r* = −0.31, *p* = 0.049; Fig. 9-1*B*). Factorial analysis revealed no effect of sex on any markers (for details, see Fig. 9-1*B*,*C*).

10.1523/JNEUROSCI.2793-17.2017.f9-1 Figure 9-1 NR4A1 protein levels in AD correlated with measures of synaptic markers and cognitive function. (A) Demographic information. F, female; M, male; FC, frontal cortex (medial anterior: Brodmann area 9–10); PMI, postmortem interval (hours); MMSE, mini mental state examination test; Medications of persons during life were not accessible (NA) as per HIPAA guidelines for a pathological study. Refers to Figure 9A. (B) Protein levels of NR4A1, AMPK and synaptic markers in PFC of AD and CTR. Data are means ± SEM of the optical density (OD) ratio between markers and GAPDH, and normalized to the CTR group. Pearson correlations between MMSE scores and protein levels in N = 17 CTR and 24 AD. Refers to Figure 9F. (C) There is no significant effect of gender on the expression of markers within CTR and AD groups. Data are means ± SEM of the OD ratio between markers and GAPDH. Refers to Figure 9F. Download Figure 9-1, TIF file

## Discussion

NR4A1 is a transcription factor that we found deregulated in PFC of animal models of stress and excessive CORT levels, and in humans in whom mental health diseases for which disrupted CORT levels and stress are established aggravating factors were diagnosed (de Quervain et al., 2004; Holsboer and Ising, 2010; Machado et al., 2014; Duman et al., 2016). Our study provides the first substantial support for an idea long speculated about. That is, dendritic spine excitatory synaptic reduction following chronic stress or disrupted CORT levels is a compensatory mechanism resulting from the overactivity of cortical networks (Popoli et al., 2011). Although synaptic attrition and reorganization might compromise cognitive functions (Sampath et al., 2017), it minimizes adverse effects that would otherwise result from the prolonged bioenergetics burden on neurons (Harris et al., 2012; Picard et al., 2014; Jeanneteau and Arango-Lievano, 2016). NR4A1 provides such a link and a mechanism for how chronic stress and chronic CORT exposure lead to dendritic spine loss in PFC. Our study demonstrated that NR4A1 uses its transcriptional activity to modify mitochondrial proton leak and dendritic spine number in cortical pyramidal neurons. Our study also provided evidence that neurotoxicity of chronic stress and CORT can be lessened by the knockdown and knockout of NR4A1 or partly counteracted by the molecular inactivation of NR4A1 with a dominant-negative approach.

### Adaptive response to protect neurons from damages of overexcitation

As an activity-dependent immediate–early gene with high inducible pattern, high turnover rate, and an option for deactivation by nuclear export, NR4A1 presents with the necessary attributes to temporarily adjust cellular fate to the triggering stimulus (Maxwell and Muscat, 2006; Helbling et al., 2014). Such cell-autonomous and time-locked expression of NR4A1 should help to contain the neuronal damage from overexcitation by tempering the reception of excitatory signals and/or the response to such excitation. We found evidence supporting both possibilities, as NR4A1 reduced the number of excitatory synapses and the mitochondrial response to glutamate. Decreasing synapse number in pyramidal neurons could favor neuronal adaptation and behavioral flexibility when levels of glutamate and CORT are excessive in PFC (Popoli et al., 2011; Chattarji et al., 2015). Stress-induced atrophy of dendritic territories in PFC corresponded with aberrant levels of mitochondria-related proteins in synaptosomes and with cognitive impairment (Lopes et al., 2017). This extends previous findings in a model of epilepsy in which NR4A1 expression protected hippocampal neurons from seizure-induced damage by rendering mitochondria more resistant to cellular stress and by reducing neuronal excitability (Zhang et al., 2009).

### From adaptation to maladaptation

NR4A1 expression in cortex and hippocampus responded to gradual acute stress but showed tolerance to chronic stress (Umemoto et al., 1994). The contrary was observed in other brain regions, indicating circuit specificity (Campos-Melo et al., 2011). This could result from differential epigenetic modifications at the *Nr4a1* locus as a function of genetic background, development, and stressors (Kember et al., 2012; Mizuno et al., 2012; Tognini et al., 2015). The subcellular distribution of NR4A1 is also affected differently by acute and chronic stress even though the last stressor is the same, which suggests a change of tune in the immediate response to the stressor. This could be due to BDNF expression, which is subjected to opposite changes on acute and chronic stress (Bath et al., 2013; Jeanneteau and Chao, 2013), as it deactivates NR4A1 by way of nuclear export, phosphorylation, and downregulation by the ubiquitin–proteasome system. Nuclear retention of NR4A1 upon chronic stress is consistent with its prolonged activity that surpassed that induced by acute stress even though NR4A1 levels were higher in PFC neurons after acute rather than chronic stress. This is consistent with the low levels of cyto-NR4A1 despite the high levels of total NR4A1 found in PFCs of human subjects in whom AD has been diagnosed. Levels of cyto-NR4A1 also correlated with measures of synaptic markers and cognitive function. This is mirrored by the decreased expression of BDNF in the human AD brain (Ginsberg et al., 2017). However, both high and low levels of NR4A1 have been linked to mental health issues as cortical levels of NR4A1 were reported to be decreased in autism and schizophrenia (Corley et al., 2016; Li et al., 2016) and NR4A1 knockouts exhibit learning disabilities (McNulty et al., 2012) as well as transgenic mice with dominant-negative NR4A1 that showed defects of synaptic plasticity (Hawk et al., 2012; Bridi and Abel, 2013).

### A mechanistic framework to study signaling loops between mitochondria and synapses

An important question raised by our study is to what extent the reduction of excitatory synapse number resulted from mitochondrial uncoupling. Manipulations of AMPK signaling established a functional link among NR4A1 transcriptional activity, AMPK, and excitatory synapses, which is corroborated by previous studies implicating AMPK in the regulation of synapse number, synaptic transmission, learning disabilities, and amyloid toxicity (Potter et al., 2010; Vingtdeux et al., 2011; Mairet-Coello et al., 2013; Ma et al., 2014). Uncoupling of mitochondrial respiration from proton transport resulted in a shortage of ATP stocks (Nicholls and Ward, 2000), activation of AMPK signaling (Weisová et al., 2012), and dendritic spine attrition (Dickey and Strack, 2011). We found that NR4A1 required its transcriptional activity to increase the proton leak in mitochondrial membranes and to increase ATP use in the context of glutamate stimulation. Previous studies showed that NR4A1 modulates mitochondria functions via genomic and nongenomic mechanisms (Close et al., 2013; Pawlak et al., 2015). Thus, NR4A1 target genes are likely important for regulating metabolic and morphological aspects of neuronal functions. NR4A1 modified the expression of several mitochondria-regulatory genes, but only the *UCPs* (*Ucp2*, *Ucp4*, and *Ucp5*) were countermodified by BDNF as it deactivated NR4A1. UCP4 dissipates transmembrane proton gradient in mitochondria to moderate respiration and to reduce reactive oxygen species damage from oxidative phosphorylation (Liu et al., 2006; Oita et al., 2009; Zhao and Bruemmer, 2010; Klotzsch et al., 2015). Previous silencing of UCP4 rendered mitochondria less resistant to glutamate excitotoxicity (Chan et al., 2006). Assuming that NR4A1-dependent induction of UCP4 could decrease the dendritic spine number in the context of chronic stress and chronic CORT exposure, our study provides a mechanistic framework for investigating whether mitochondrial uncoupling could serve as a rheostat to isolate mitochondrial respiration from excessive excitatory stimulation that would endanger neuronal survival.

### Methodological limitations

NR4A1 activity was not measured directly in human cortical tissues because it was not possible in postmortem biopsy samples. The monitoring of p-NR4A1 could have strengthened the other readouts (total protein or RNA levels and cyto-NR4A1) that only reflect NR4A1 activity indirectly. But we did not test for p-NR4A1 in human PFC tissues, as it could be equivocal in postmortem analysis. Previous study of fresh fibrotic tissues indicated that p-NR4A1 was inversely correlated with total NR4A1 levels and activity (Palumbo-Zerr et al., 2015), which is consistent with our findings in the human brain. The effect of medication on NR4A1 expression and activity is possible, but prescription medications were not disclosed as per HIPAA policies for a pathological study. The drugs used to manipulate AMPK activity provided only mild modulatory effects. But there are no direct inhibitors of AMPK that would suppress its activity or, currently, other activators specific and more potent than A769662 (Steinberg and Kemp, 2009; Scott et al., 2014). The magnitude of stress and CORT-mediated attrition of dendritic spines depends on age (Bloss et al., 2011; McEwen and Morrison, 2013). Our study investigated the PFCs of adolescent animals guided by previous reports in rodents of similar age subjected to similar procedures (Gourley et al., 2013; Swanson et al., 2013; Anderson et al., 2016). In particular, our studies were conducted on various animal species (KO mice and KO rats) and strains (C57BL/6 and CD1) by distinct investigators. Despite the heterogeneity of the models, knock-out, knock-down, and dominant-negative approaches all showed a role of NR4A1 in activating AMPK and reducing excitatory synapses number in cortex. The *in utero* electroporation system offered the advantages of unilateral targeting of sparse cortical neurons during embryogenesis, but impact on behavior was not informative. In contrast, constitutive knockouts exhibited a behavioral phenotype and permitted cross-validation of biochemical and morphological findings. Yet, NR4A1 is expressed in many cell types, which could bias the interpretations of KO data (Hawk and Abel, 2011; Safe et al., 2016). For instance, we found discrepancies in the effects of NR4A1 KO compared with its knockdown on dendritic spine number in PFC. Importantly, the hypothalamic–pituitary–adrenal axis signaling was normal in NR4A1-deficient mice, indicating that CORT secretion and response are not biased in this model (Crawford et al., 1995).

### Perspectives

There is no known endogenous ligand for NR4A1 but synthetic ligands with agonistic and antagonistic properties are being developed to regulate cellular fate in a variety of disease models (Safe et al., 2016). Drug-induced activation of NR4A1 to compensate for its low expression in a model of autism reduced the number of surplus synapses offering perspectives for therapeutic intervention (Li et al., 2016). There are also opportunities for clinical applications of NR4A1 ligands that would promote NR4A1 downregulation, nuclear export, or transcriptional blockade.

*Note Added in Proof:* One of the grants funding the work was accidentally not listed in the Early Release version published January 2, 2018. The funding information has now been corrected.
