# Supplementary figures and images for: The Stress-Induced Transcription Factor NR4A1 Adjusts Mitochondrial Function and Synapse Number in Prefrontal Cortex
Source: J Neurosci. 2018 Feb 7;38(6):1335–50. doi: 10.1523/JNEUROSCI.2793-17.2017 (PMC5815341; doi:10.1523/JNEUROSCI.2793-17.2017)

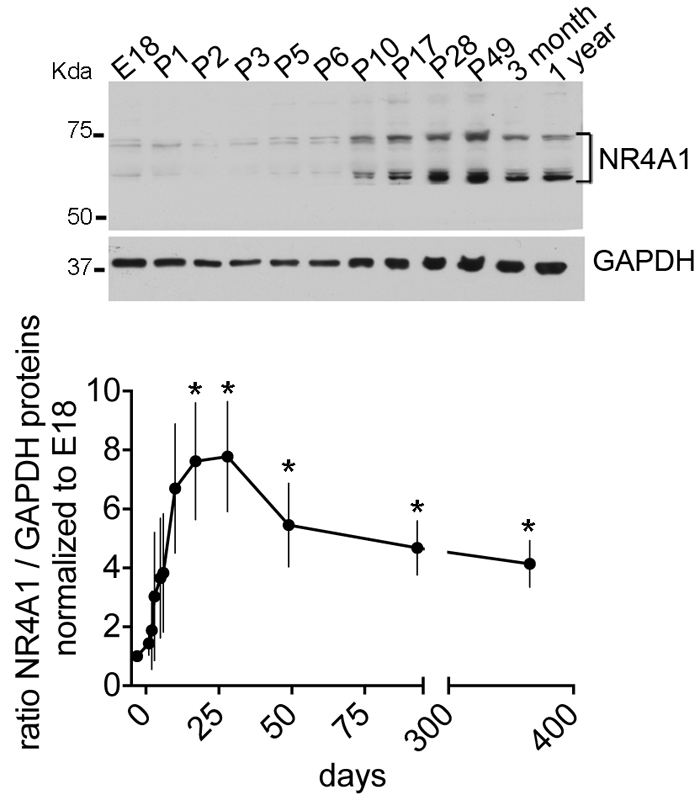

Supplement: Figure 1-1 [file zns999180478so1.tif]

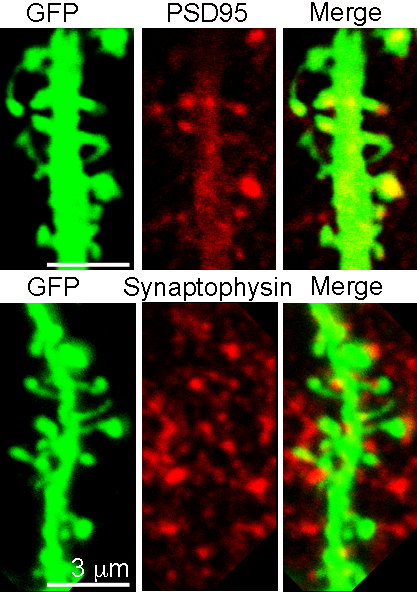

Supplement: Figure 1-2 [file zns999180478so2.tif]

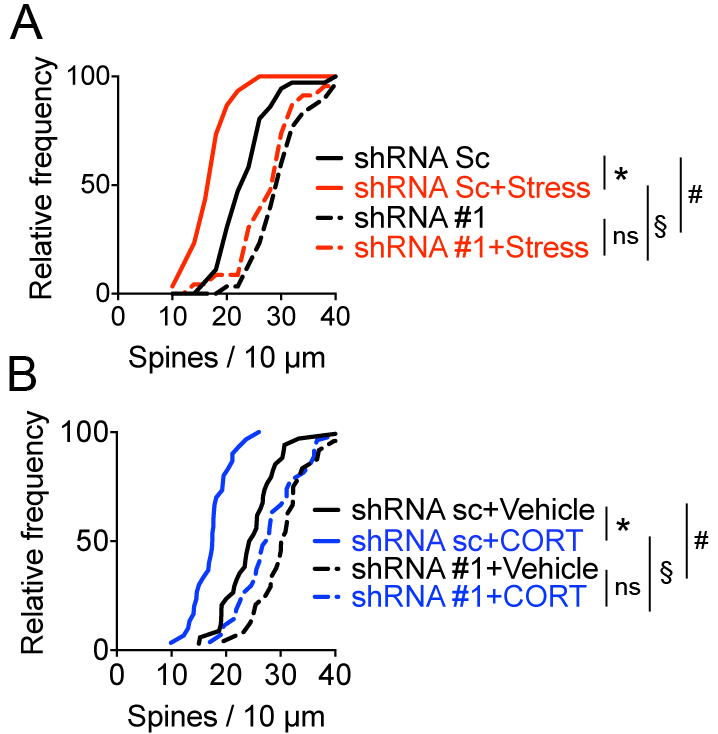

Supplement: Figure 1-3 [file zns999180478so3.tif]

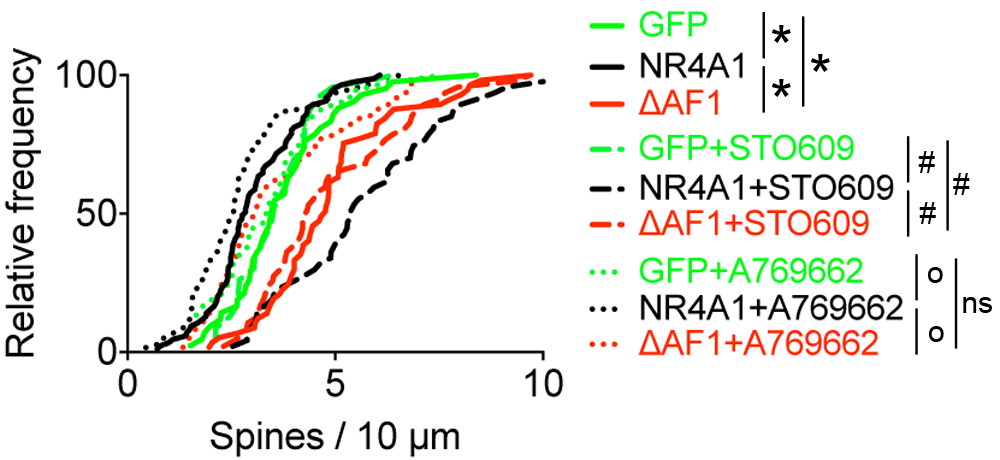

Supplement: Figure 6-1 [file zns999180478so4.tif]

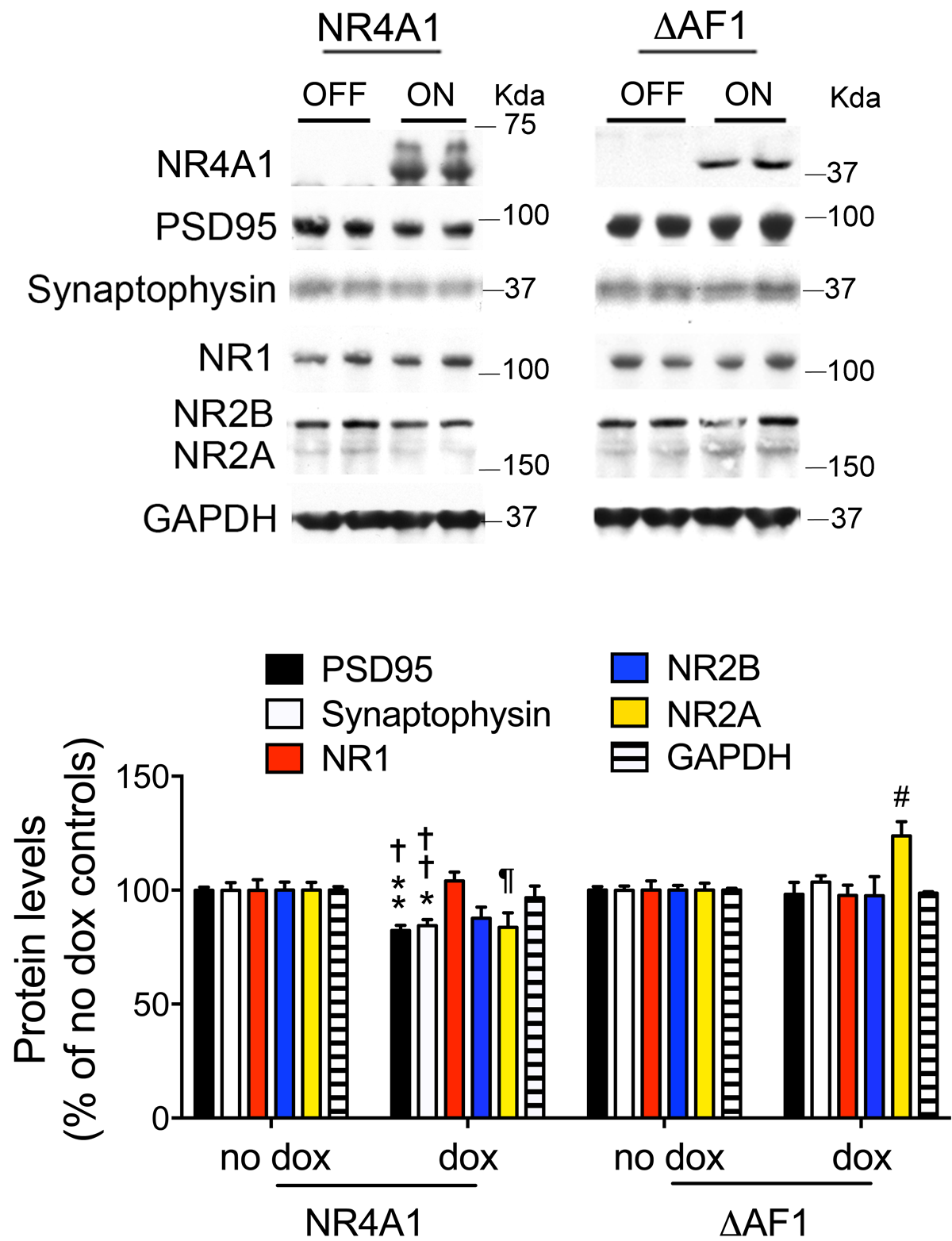

Supplement: Figure 7-1 [file zns999180478so5.tif]

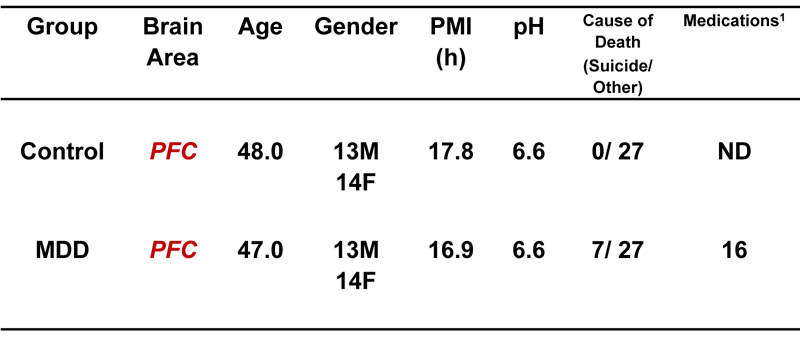

Supplement: Figure 8-1 [file zns999180478so6.tif]

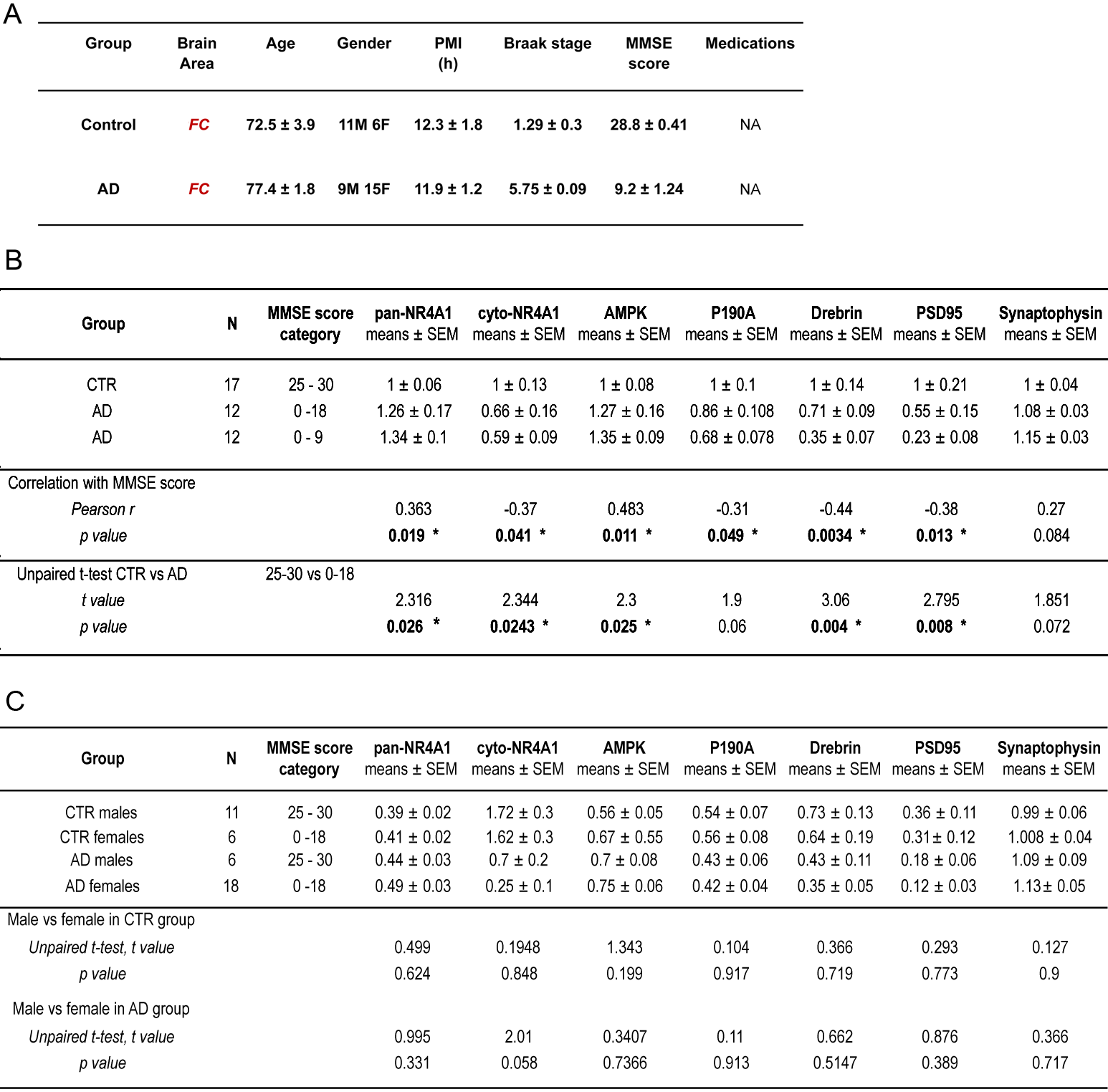

Supplement: Figure 9-1 [file zns999180478so7.tif]
